# The Generalized Method of Wavelet Moments with eXogenous inputs: a fast approach for the analysis of GNSS position time series

**DOI:** 10.1007/s00190-023-01702-8

**Published:** 2023-02-06

**Authors:** Davide A. Cucci, Lionel Voirol, Gaël Kermarrec, Jean-Philippe Montillet, Stéphane Guerrier

**Affiliations:** 1 Geneva School of Economics and Management, University of Geneva, Geneva, Switzerland; 2 Institute for Meteorology and Climatology, Leibniz University Hannover, Hannover, Germany; 3 Institute Dom Luiz (IDL), University of Beira Interior, Covilhã, Portugal; 4 Physikalisch-Meteorologisches Observatorium Davos/World Radiation Center (PMOD/WRC), Davos, Switzerland; 5 Faculty of Science, University of Geneva, Geneva, Switzerland

**Keywords:** Maximum likelihood estimator, Variance decomposition, Two-step estimation, Long-range dependence, Tectonic, Geodynamics

## Abstract

The global navigation satellite system (GNSS) daily position time series are often described as the sum of stochastic processes and geophysical signals which allow to study global and local geodynamical effects such as plate tectonics, earthquakes, or ground water variations. In this work, we propose to extend the Generalized Method of Wavelet Moments (GMWM) to estimate the parameters of linear models with correlated residuals. This statistical inferential framework is applied to GNSS daily position time-series data to jointly estimate functional (geophysical) as well as stochastic noise models. Our method is called GMWMX, with X standing for eXogenous variables: it is semi-parametric, computationally efficient and scalable. Unlike standard methods such as the widely used maximum likelihood estimator (MLE), our methodology offers statistical guarantees, such as consistency and asymptotic normality, without relying on strong parametric assumptions. At the Gaussian model, our results (theoretical and obtained in simulations) show that the estimated parameters are similar to the ones obtained with the MLE. The computational performances of our approach have important practical implications. Indeed, the estimation of the parameters of large networks of thousands of GNSS stations (some of them being recorded over several decades) quickly becomes computationally prohibitive. Compared to standard likelihood-based methods, the GMWMX has a considerably reduced algorithmic complexity of order $\mathcal {O}\{\log (n) n\}$ for a time series of length *n*. Thus, the GMWMX appears to provide a reduction in processing time of a factor of 10–1000 compared to likelihood-based methods depending on the considered stochastic model, the length of the time series and the amount of missing data. As a consequence, the proposed method allows the estimation of large-scale problems within minutes on a standard computer. We validate the performances of our method via Monte Carlo simulations by generating GNSS daily position time series with missing observations and we consider composite stochastic noise models including processes presenting long-range dependence such as power law or Matérn processes. The advantages of our method are also illustrated using real time series from GNSS stations located in the Eastern part of the USA.

## Introduction

Permanent stations observing signals from the Global Navigation Satellite System (GNSS) have been installed worldwide. They measure changes in position over time associated with a number of geophysical phenomena such as post-glacial rebound (see, e.g.,  Milne et al. 2001), hydrological loading (see, e.g., Bevis et al. 2002; Tregoning and Watson 2009) or crustal deformations (see, e.g., Williams 2003), with millimeter-level accuracy. These geophysical signals can be studied through a careful analysis of daily time series of GNSS receiver coordinates (Bock and Melgar 2016; Herring et al. 2016; He et al. 2017). Many applications focus on the estimation of the tectonic rate (Bock et al. 1997; Bock and Melgar 2016) either as a linear function (Fernandes et al. 2004; Bos et al. 2020), or as a nonlinear trend including offsets (Nielsen et al. 2013; Blewitt et al. 2016). To that end, the daily position time series are described as the sum of a noise and a geophysical signal. The latter can again be divided into station displacements due to geophysical phenomena (e.g., seasonal variations, tectonic movements, post-seismic relaxations of the crust) and other factors (e.g., small amplitude transient signals due to various disturbances He et al. 2017; Michel et al. 2021).

Bevis and Brown (2014) are the first to suggest that the equations used to describe the motion of GNSS stations should be thought of as functional (or trajectory) models. This approach has also been applied to various fields such as gravity time series (Van Camp et al. 2005), mean sea-level records (Burgette et al. 2013; Montillet et al. 2020), and bridge oscillations (Omidalizarandi et al. 2020). In this contribution, we follow Bevis and Brown (2014) and He et al. (2019) and describe the geodetic time series by a functional and a stochastic noise model. We focus on obtaining the suitable parameter estimates together with reasonable associated uncertainties (Langbein 2008; Teferle et al. 2008; Bos et al. 2010; He et al. 2019; Bevis et al. 2020; He et al. 2021). The joint estimation of both deterministic and stochastic models is often based on the maximum likelihood estimator (MLE) and has been implemented in various software packages such as CATS (Williams 2008), Est_noise (Langbein 2008) and Hector (Bos et al. 2008). Other methods use the Markov chain Monte Carlo (Olivares and Teferle 2013) or the expectation-maximization (EM) algorithm (Kargoll et al. 2020).

Unfortunately, the computational aspects related to the parameter estimation are often a key challenge when considering large datasets and/or complex stochastic noise models. Generally, various matrix operations are needed to compute the likelihood function which can become rapidly cumbersome for longer and longer time series. Powerful computing facilities (e.g., parallel processing, national computing centers) are required in order to process hundreds of stations, with some of them recording observations over several decades, in a reasonable amount of time. To speed up the processing time, several approximations of the MLE have been proposed. Bos et al. (2008, 2013) reduced the computation time of a factor of 10–100 compared to the standard MLE method (depending on the length of the real time series) initially developed by Williams (2008). Tehranchi et al. (2021) further improved the computational aspect of the method using restricted MLE. Despite these computational improvements, the analysis of crustal deformation or geodynamical activity on a large scale that (i) includes hundreds to thousands of GNSS stations (He et al. 2021), (ii) with some of them recording more than 25 years of continuous observations and (iii) when different noise models must be tested, becomes impractical due to the large amount (e.g., weeks) of processing time required (He et al. 2019; Bos et al. 2020).

In this contribution, we propose a semi-parametric, computationally efficient and scalable method to estimate the parameters of linear models with dependent residuals. The key advancement of this new approach is that it avoids the use of strong parametric assumptions and drastically reduces the computational time required to estimate the models commonly used to describe GNSS time-series data. Our method relies on a two-step statistical procedure which considers a (weighted) least squares approach to estimate the functional part of the model while the stochastic part of the model is obtained using the Generalized Method of Wavelet Moments (GMWM) proposed in Guerrier et al. (2013). We call our method the Generalized Method of Wavelet Moments with eXogenous inputs, or GMWMX. Interestingly, the Least Squares Variance Component Estimation (LS-VCE) proposed in Pukelsheim (1976) (as well as independently discovered and popularized in Geodesy by Teunissen 2004) and further developed and elaborated by Teunissen and Amiri-Simkooei (2008) and Amiri-Simkooei (2007) is related to the proposed approach. Indeed, the LS-VCE is also a moment-based semi-parametric method with desirable computational properties.

We test our method against the MLE using the Hector package developed by Bos et al. (2008) and He et al. (2019), a standard software to analyze geodetic time series. We focus especially on the processing time as a function of the length of the time series and the accuracy of the estimated geophysical parameters considering different stochastic noise models (e.g., a combination of power law and white noise). Our analysis includes simulated and real GNSS daily position coordinate time series. The real data are provided by a few selected GNSS stations located on the east coast of the USA. We compare our estimates with (i) Hector’s solutions and (ii) the velocity estimates provided by the Plate Boundary Observatory (PBO - UNAVCO) (Herring et al. 2016).

This paper is organized as follows: The next section introduces the mathematical notations and a summary of the MLE. Section 3 derives the new estimator and discusses the contribution of the GMWMX with a specific application to GNSS daily position time-series analysis. We then compare the results of our new estimator to the one obtained with the Hector software package for simulated and real observations in Sects. 4.1 and 4.2, respectively. We conclude with a discussion on the use of the GMWMX in environmental geodesy in Sect. 5.

## Problem formulation

### Generalities and notations

Throughout this paper, we employ the following notations. For a vector $\textbf{a} \in {\mathbb {R}}^n$, we define $\textbf{a}_{i}$ as *i*-th element of the vector $\textbf{a}$, for $i = 1, \ldots , n$. Similarly, for a matrix $\textbf{A} \in {\mathbb {R}}^{n \times m}$, we define $\textbf{A}_{i,j}$ as the (*i*, *j*)-th element of the matrix $\textbf{A}$, for $i = 1, \ldots , n$ and $j = 1, \ldots , m$, and we denote $\textbf{A}_i$ as the *i*-th row of $\textbf{A}$. Given two matrices $\textbf{A}, \, \textbf{B} \in {\mathbb {R}}^{n \times m}$, we write $\textbf{A} \, \propto \, \textbf{B}$ to denote that $\textbf{A}$ is proportional to $\textbf{B}$ in the sense that there is a nonzero constant *k* such that $\textbf{A} = k \textbf{B}$. Similarly, we write  to denote that $\textbf{A}$ is not proportional to $\textbf{B}$. Moreover, we write $\textbf{A} > 0$ and $\textbf{A} \geqslant 0$ to denote that the matrix $\textbf{A}$ is positive definite and positive semi-definite, respectively. Finally, we use the notation $\overset{p}{\longrightarrow }$ and $\overset{d}{\longrightarrow }$ to denote convergence in probability and in distribution, respectively.

This work aims at developing a statistical inferential framework for the parameters of linear regression models with correlated residuals. While the method proposed in this article is generally applicable to various regression problems we consider in particular the models used for GNSS (daily) position time series. More precisely, we assume that the observations are generated from the following model:

1 $$\begin{aligned} \textbf{Y} = \textbf{A} {\textbf{x}}_0 + \varvec{\varepsilon }, \end{aligned}$$

where $\textbf{Y} \in {\mathbb {R}}^n$ denotes the response variable of interest (i.e., a vector of GNSS observations), $\textbf{A} \in {\mathbb {R}}^{n \times p}$ denotes a fixed design matrix, ${\textbf{x}}_0 \in \mathcal {X} \subset {\mathbb {R}}^p$ denotes a vector of unknown constants and $\varvec{\varepsilon } \in {\mathbb {R}}^n$ a vector of (zero mean) residuals. In many applications, ${\textbf{x}}_0$ is of interest as it is related to, for example, the local tectonic rate and seismic phenomena (see, e.g., Bock and Melgar 2016). A common formulation of the functional component of the model is given by He et al. (2017), which expresses the *i*-th component of the vector $\textbf{A} {\textbf{x}}_0$ as follows:

2 $$\begin{aligned}{} & {} \mathbb {E}[\textbf{Y}_i] = \textbf{A}_i {\textbf{x}}_0 = a+b\left( t_{i}-t_{0}\right) \nonumber \\ {}{} & {} +\sum _{h=1}^{2}\left[ c_{h} \sin \left( 2 \pi f_{h} t_{i}\right) +d_{h} \cos \left( 2 \pi f_{h} t_{i}\right) \right] \nonumber \\{} & {} + \sum _{k=1}^{n_{g}} g_{k} H\left( t_{i}-t_{k}\right) , \end{aligned}$$

where *a* is the initial position at the reference epoch $t_0$, *b* is the velocity parameter, $c_k$ and $d_k$ are the periodic motion parameters ($h = 1$ and $h = 2$ represent the annual and semi-annual seasonal terms, respectively). The offset terms model earthquakes, equipment changes or human intervention in which $g_k$ is the magnitude of the change at epochs $t_k$, $n_g$ is the total number of offsets, and *H* is the Heaviside step function. Moreover, we assume that $\varvec{\varepsilon }_i=\textbf{Y}_i-\mathbb {E}[\textbf{Y}_i]$ is a strictly (intrinsically) stationary process and that

3 $$\begin{aligned} \varvec{\varepsilon } \sim \mathcal {F} \left\{ \textbf{0}, \varvec{\Sigma }(\varvec{\gamma }_0)\right\} , \end{aligned}$$

where $\mathcal {F}$ denotes some probability distribution in ${\mathbb {R}}^n$ with mean $\textbf{0}$ and covariance $\varvec{\Sigma }(\varvec{\gamma }_0)$. We assume that $\varvec{\Sigma }(\varvec{\gamma }_0) > 0$ and that it depends on the unknown parameter vector $\varvec{\gamma }_0 \in \varvec{\Gamma } \subset {\mathbb {R}}^q$. This parameter vector specifies the covariance of the observations and is often referred to as the *stochastic parameters*. The formulation of the noise structure of $\varvec{\varepsilon }$ is very general and includes a large class of time series models such as (the sum of) AutoRegressive Moving-Average (ARMA) models with additional noise, rounding errors and/or processes with long-range dependence. For example, this class of models includes the model considered in He et al. (2017) by assuming $\mathcal {F}$ to be a multivariate normal distribution and that $\varepsilon _t = Z_t + R_t + U_t$, where $Z_t$ represents a Matérn process (see, e.g., Lilly et al. 2017), $R_t$ denotes a fractional (Gaussian) noise (see, e.g., Li and Lim 2006) and $U_t$ represents a standard Gaussian white noise process. In practice, the estimation of $\varvec{\gamma }_0$ is of interest as it could be informative regarding soil properties, such as moisture and groundwater depletion (see, e.g., Bevis et al. 2005), as well as atmospheric properties, which are of importance in climate change studies (Wöppelmann et al. 2009).

For simplicity, we let $\varvec{\theta }_0 = \left[ \varvec{\textbf{x}}_0^{\textrm{T}}\;\; \varvec{\gamma }_0^{\textrm{T}}\right] ^{\textrm{T}}\in \varvec{\Theta } = \mathcal {X} \times \varvec{\Gamma } \subset {\mathbb {R}}^{p + k}$ denote the unknown parameter vector of the model described in (1). The main goal of this paper is to propose a *computationally efficient* inferential framework for $\varvec{\theta }_0$ which enjoys desirable statistical properties while avoiding the specification of the probability distribution $\mathcal {F}$. Throughout this paper, we consider a general class of probability distributions $\mathcal {F}$, which can be characterized by a set of mild regularity conditions specified later in Sect. 3.

### Standard likelihood-based approach

The standard approach for the estimation of the problem defined in (1) is based on the MLE (see, e.g., Bos et al. 2008) or closely related estimators such as the restricted MLE (see, e.g., Tehranchi et al. 2021). In this section, we briefly review how maximum likelihood estimators can be constructed in this setting. Under the parametric assumption that the probability distribution $\mathcal {F}$ considered in (3) is a multivariate normal distribution, the likelihood function for a generic $\varvec{\theta } \in \varvec{\Theta }$ is simply given by:

4 $$\begin{aligned}{} & {} L\left( \varvec{\theta } | \textbf{Y} \right) = \exp \Bigg \{-\frac{1}{2} \left( \textbf{Y} - \textbf{A}\textbf{x}\right) \nonumber \\{} & {} \quad ^{\textrm{T}}{\varvec{\Sigma }\left( \varvec{\gamma }\right) } ^{-1}\left( \textbf{Y}-\textbf{A}\textbf{x}\right) \Bigg \} \left[ (2\pi )^n \det \left\{ \varvec{\Sigma }\left( \varvec{\gamma }\right) \right\} \right] ^{-1/2}, \end{aligned}$$

allowing to define the MLE for $\varvec{\theta }_0$ as

5 $$\begin{aligned} \widehat{\varvec{\theta }} = \left[ \widehat{\textbf{x}}^{\textrm{T}}\;\; \widehat{\varvec{\gamma }}^{\textrm{T}}\right] ^{\textrm{T}}= \underset{\varvec{\theta } \in \varvec{\Theta }}{{{\,\textrm{argmax}\,}}}\; L\left( \varvec{{\varvec{\theta }}} | \textbf{Y} \right) . \end{aligned}$$

Under standard regularity conditions (see, e.g., Newey and McFadden 1994), this estimator enjoys some desirable statistical properties such as consistency and asymptotic normality. In particular, under usual smoothness and mixing conditions it can be shown that

6 $$\begin{aligned}{} & {} \sqrt{a_n}\left( \widehat{\textbf{x}} - \textbf{x}_0 \right) \overset{{d}}{\longrightarrow } \mathcal {N}\left( \textbf{0}, \textbf{V} \right) , \;\;\; \nonumber \\{} & {} \quad \text {where} \;\;\; \textbf{V} = \lim _{n \rightarrow \infty } a_n \; \left\{ \textbf{A}^{\textrm{T}}\varvec{\Sigma }(\varvec{\gamma }_0)^{-1} \textbf{A} \right\} ^{-1} , \end{aligned}$$

where $\{a_n\}_{n \in \mathbb {N}}$ is a diverging sequence of positive numbers such that $\sqrt{a_n}$ corresponds to the asymptotic rate of convergence of $\widehat{\textbf{x}}$. In the case of short-memory processes, we typically have $a_n = n$ and $\widehat{\textbf{x}}$ is a $\sqrt{n}$-consistent estimator. However, slower rates of convergence occur with long-memory processes such as the ones typically considered to model GNSS (daily) position time series (see, e.g., Palma 2007 and the references therein for more details). Moreover, the result presented in (6) relies on the assumption that $n^{-1}\textbf{A}^{\textrm{T}}\textbf{A}$ converges (possibly in probability in the case of a random design) to a nonsingular matrix $\textbf{Q} \in {\mathbb {R}}^{p \times p}$. Thus, the matrix $\textbf{A}$ defined in (1) may have to be re-scaled appropriately to ensure that there exists a common asymptotic rate of convergence $\sqrt{a_n}$ for all the elements of $\widehat{\textbf{x}}$ (see, e.g., Chapter 16 of Hamilton 1994 and the references therein for more details). This assumption on the matrix $\textbf{A}$ is made throughout the paper.

The estimator $\widehat{\textbf{x}}$ is *asymptotically efficient* since Aitken’s theorem (or more precisely its generalization given in Hansen 2022) shows that, in general, *any* unbiased estimator of $\textbf{x}$ (even in the case where $\varvec{\Sigma }(\varvec{\gamma }_0)$ is known), say $\bar{\textbf{x}}$, is such that

7 $$\begin{aligned} \lim _{n \rightarrow \infty } \; a_n {{\,\textrm{var}\,}}\left( \bar{\textbf{x}}\right) \geqslant a_n \left\{ \textbf{A}^{\textrm{T}}\varvec{\Sigma }(\varvec{\gamma }_0)^{-1} \textbf{A} \right\} ^{-1}. \end{aligned}$$

Thus, the estimator $\widehat{\textbf{x}}$ is said to be asymptotically efficient in the sense that its asymptotic variance is the smallest possible among all unbiased estimators.

An important limitation of the MLE is the computational burden it often entails. Indeed, solving (5) typically requires to evaluate the likelihood function in (4) a large number of times. Each evaluation involves the inversion of the $n \times n$ matrix $\varvec{\Sigma }(\varvec{\gamma }_0)$ in (4), which is computationally expensive and can become problematic when considering large sample sizes.

Alternatively, the Kalman filter can be used together with the EM algorithm to compute $\widehat{\varvec{\theta }}$ while avoiding the matrix operations presented in (4) (see Dempster et al. 1977; Shumway and Stoffer 1982; Shumway et al. 2000). While this approach can provide a viable solution in some cases, the “M” step can be very complex, while the “E” step is often computationally cumbersome; therefore, finding the MLE is not always a simple task. Moreover, this approach becomes particularly challenging when *n* is large and/or when the model describing $\varepsilon _i$ is complex such as a sum of latent random processes as presented, for example, in Sect. 2.1. The limited practical applicability of the MLE in this context is, for example, illustrated in Stebler et al. (2014).

Furthermore, the MLE presented in this section and considered, for example, in Bos et al. (2020) is based on strong parametric assumptions (often referred to as the Gauss–Markov hypothesis) that the noise $\varvec{\varepsilon }$ follows a multivariate Gaussian distribution. These assumptions are often difficult to verify in practice and often appear unrealistic due to the presence of large mean deviations in GNSS time series.

## The Generalized Method of Wavelet Moments with eXogenous inputs

**Fig. 1:**
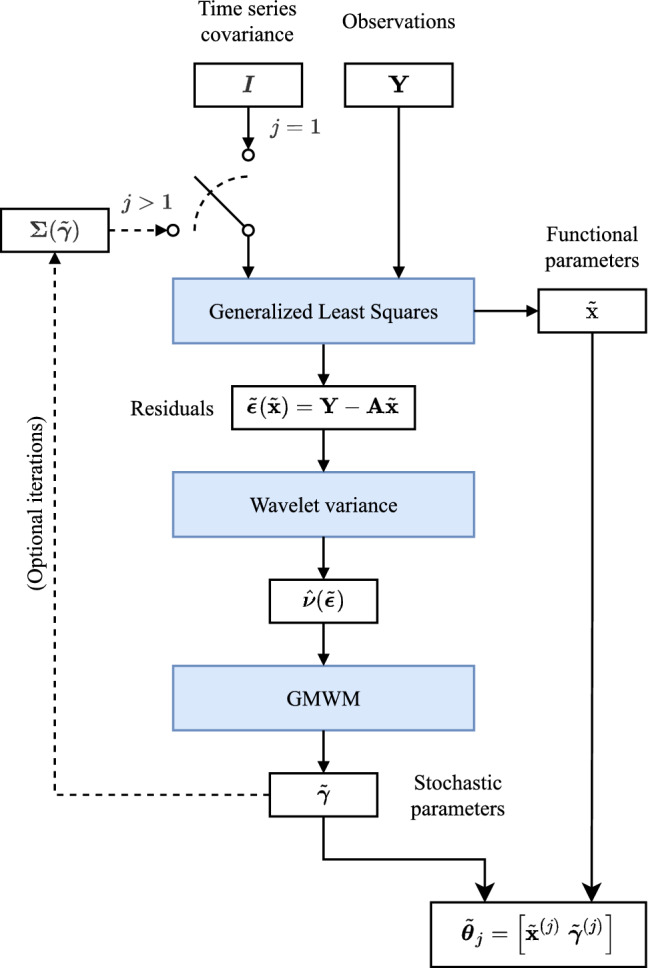
Flowchart representing the GMWMX method described in Sect. 3.1

In this section, we introduce the GMWMX approach which extends the standard GMWM of Guerrier et al. (2013) in the context of linear regression with correlated residuals. This method can be applied, for example, to the estimation of the parameters of the model described in (1). The proposed approach is computationally efficient and allows to considerably alleviate the computational limitations of standard methods such as the MLE. Unlike methods relying on a fully specified parametric model, we relax some of the requirements imposed on $\mathcal {F}$. Indeed, we only require that $\varvec{\varepsilon }_t$ is a strictly (intrinsically) stationary process with finite fourth moment and covariance matrix $\varvec{\Sigma }(\varvec{\gamma }_0)$. Thus, the GMWMX is a *semi-parametric* method in the sense that its statistical properties are preserved for a general class of probability distributions $\mathcal {F}$ (which can remain unspecified). Compared to fully parametric methods such as the MLE, our approach provides statistical guarantees *for all* zero-mean probability distributions (with finite fourth moment) and covariance matrix $\varvec{\Sigma }(\varvec{\gamma }_0)$. Moreover, the GMWMX is a semi-parametric approach based on the principle of generalized least squares (GLS) combined with the GMWM framework. Indeed, our approach considers initially a coarse approximation of $\varvec{\Sigma } (\varvec{\gamma }_0)$ as defined in (3), which is used in a GLS approach to obtain an estimate of $\textbf{x}_0$. From this estimate, we then compute a GMWM-based estimator of $\varvec{\gamma }_0$. Our framework allows to iterate this process in order to improve statistical efficiency. The procedure is schematically depicted in Fig. 1, formally defined in Sect. 3.1, and its benefits are summarized in Sect. 3.2.

### Proposed statistical framework

The GLS is a common method used to estimate the unknown parameters in a linear regression model with correlated residuals, assuming that the covariance matrix $\varvec{\Sigma }(\varvec{\gamma }_0)$ of $\varvec{\varepsilon }$ is known. In our setting, this requirement is not realistic, but we let $\varvec{\Sigma }$ denote the *assumed* covariance matrix of $\varvec{\varepsilon }$. The notation $\varvec{\Sigma }$ is used to highlight that ${\varvec{\gamma }}_0$ (and thus $\varvec{\Sigma }({\varvec{\gamma }}_0)$) is unknown allowing to consider different approximations of $\varvec{\Sigma }({\varvec{\gamma }}_0)$ by $\varvec{\Sigma }$. Based on the assumed covariance $\varvec{\Sigma }$, we obtain the following GLS estimator:

8 $$\begin{aligned} {\widetilde{{\textbf {x}}}}\left( \varvec{\Sigma }\right){} & {} = \, \underset{{\textbf {x}}\in \mathcal {X}}{{{\, \text {argmin}\,}}}\; \left\{ {\textbf {Y}}- {\textbf {A}}{} {\textbf {x}}\right\} ^{\text {T}} \varvec{\Sigma }^{-1} \left\{ {\textbf {Y}}- {\textbf {A}}{} {\textbf {x}}\right\} \nonumber \\{} & {} = \left( {\textbf {A}}^{\text {T}}\varvec{\Sigma }^{-1} {\textbf {A}}\right) ^{-1} {\textbf {A}}^{\text {T}}\varvec{\Sigma }^{-1} {\textbf {Y}}. \end{aligned}$$

In the case where we consider the crude approximation $\varvec{\Sigma }\, \propto \, \textbf{I}$, the estimator reduces to the ordinary least squares estimator and we obtain:

9 $$\begin{aligned}{} & {} {\widetilde{\textbf{x}}} = {\widetilde{\textbf{x}}}\left( \textbf{I}\right) = \left( \textbf{A}^{\textrm{T}}\textbf{A}\right) ^{-1} \textbf{A}^{\textrm{T}}\textbf{Y}. \end{aligned}$$

This estimator is simple to compute and enjoys well-known statistical properties (as discussed later in this section). Indeed, under very mild conditions based on functional dependence measures as proposed initially by Wu (2005), we have that ${\widetilde{\textbf{x}}}$ is consistent for $\textbf{x}_0$ and admits the following limiting distribution (see Theorem 1 of Wu 2007):

10 $$\begin{aligned}{} & {} \sqrt{a_n} \left( {\widetilde{\textbf{x}}} - \textbf{x}_0 \right) \overset{d}{\longrightarrow }\ \mathcal {N}\left( \textbf{0}, \textbf{V}^{*} \right) , \nonumber \\{} & {} \quad \text {where} \;\;\; \textbf{V}^{*} = \lim _{n \rightarrow \infty } a_n \left( \textbf{A}^{\textrm{T}}\textbf{A} \right) ^{-1} \textbf{A}^{\textrm{T}}\varvec{\Sigma }(\varvec{\gamma }_0) \textbf{A} \left( \textbf{A}^{\textrm{T}}\textbf{A} \right) ^{-1}. \nonumber \\ \end{aligned}$$

However, the estimator is not asymptotically efficient as compared to the MLE $\widehat{\varvec{\textbf{x}}}$, for  we have

11 $$\begin{aligned}{} & {} \lim _{n \rightarrow \infty } \; {{\,\textrm{var}\,}}\left\{ \sqrt{a_n} \left( \widetilde{\varvec{\textbf{x}}} - \varvec{\textbf{x}}_0 \right) \right\} - {{\,\textrm{var}\,}}\left\{ \sqrt{a_n} \left( \widehat{\varvec{\textbf{x}}} - \varvec{\textbf{x}}_0 \right) \right\} \nonumber \\{} & {} \quad = \textbf{V}^{*} - \textbf{V} > 0. \end{aligned}$$

The derivation of this result is given in Appendix A.1. This result implies that any linear combination of $\widetilde{\textbf{x}}$ has a larger asymptotic variance with respect to the same linear combination of $\widehat{\textbf{x}}$. Therefore, $\widetilde{\varvec{\textbf{x}}}$ is asymptotically less efficient than the MLE $\widehat{\varvec{\textbf{x}}}$ in the case of correlated and/or heteroscedastic residuals (i.e., ).

Based on a suitable estimator of $\textbf{x}_0$, such as $\widetilde{\varvec{\textbf{x}}}$, we can compute the (estimated) residuals of model (1) whose population-level version of $\varvec{\varepsilon }$ is defined as:

12 $$\begin{aligned} {\varvec{\varepsilon }}\left( \textbf{x}\right) = \textbf{Y} - \textbf{A}\textbf{x}, \end{aligned}$$

and a natural estimator of $\varvec{\varepsilon }$ is simply ${\widetilde{\varvec{\varepsilon }}} = \varvec{\varepsilon }\left( \widetilde{\textbf{x}}\right)$. This estimator is consistent for $\varvec{\varepsilon }(\textbf{x}_0)$ since $\widetilde{\textbf{x}}$ is consistent for $\textbf{x}_0$ as implied by (10) and the continuous mapping theorem. More precisely, we have for all $i \in \{1, \ldots , n\}$

$$\begin{aligned}{} & {} {\widetilde{\varvec{\varepsilon }}}_i = \varvec{\varepsilon }_i\left( \widetilde{\textbf{x}}\right) = \textbf{Y}_i - \textbf{A}_i^{\textrm{T}}\widetilde{\textbf{x}} \overset{p}{\longrightarrow }\ \textbf{Y}_i - \textbf{A}_i^{\textrm{T}}{\textbf{x}_0} = \varvec{\varepsilon }_i(\textbf{x}_0) = \varvec{\varepsilon }_i. \end{aligned}$$

The vector of residuals ${\widetilde{\varvec{\varepsilon }}}$ allows to construct an estimator of ${\varvec{\gamma }}_0$ using the GMWM methodology. The latter is an estimation framework that allows to consider a wide range of models including some complex (latent) models where standard methods typically fail due to the model complexity and/or the unrealistic computational burden they entail (see, e.g., Stebler et al. 2011, 2014). In short, this approach uses a quantity called wavelet variance (WV) (see, e.g., Percival and Walden 2000) in the spirit of a generalized method of moments (GMM) estimator of Hansen (1982). The GMWM estimator based on an estimator of $\textbf{x}_0$, say $\textbf{x}$, is defined as follows:

13 $$\begin{aligned}{} & {} \widetilde{\varvec{\gamma }}\left( \textbf{x}\right) = \underset{\varvec{\gamma } \in \varvec{\Gamma }}{{{\,\textrm{argmin}\,}}}\;\left\{ \widehat{\varvec{\nu }}\left( {\textbf{x}}\right) - \varvec{\nu }(\varvec{\gamma })\right\} ^{\textrm{T}}\varvec{\Omega } \left\{ \widehat{\varvec{\nu }}\left( {\textbf{x}}\right) - \varvec{\nu }(\varvec{\gamma })\right\} , \end{aligned}$$

where $\varvec{\nu }(\varvec{\gamma })$ is the WV vector implied by the model. This quantity is an explicit function of the parameters for a large class of models based on the general results of Zhang (2008). The vector $\widehat{\varvec{\nu }}\left( {\textbf{x}}\right)$ denotes the estimated Haar WV computed on ${\varvec{\varepsilon }}\left( {\textbf{x}}\right)$ and $\varvec{\Omega }$ corresponds to an appropriate (possibly estimated) positive-definite weighting matrix (see, e.g., Guerrier et al. 2013 for more details). Additional details on these quantities are given in Appendix B. Using the previously defined quantities, the idea behind the GMWM estimator presented in (13) is to match $\widehat{\varvec{\nu }}\left( {\textbf{x}}\right)$ with $\varvec{\nu }(\varvec{\gamma })$ within a classical minimum distance (CMD) approach. Indeed, CMD estimators exploit the mapping between the theoretical function $\varvec{\nu }(\varvec{\gamma })$ and the empirical “reduced form” quantity $\widehat{\varvec{\nu }}\left( {\textbf{x}}\right)$ in order to estimate a vector of parameters of interest (i.e., $\varvec{\gamma }_0$). While CMD estimators are (under standard regularity requirements) consistent and asymptotically normal, they are generally not statistically efficient when compared to the MLE because they consider a simpler and less informative objective function than the likelihood function (see, e.g., McFadden 1989). However, this simplification permits to substantially reduce the computational complexity of the optimization problem. In addition, this approach can avoid the full specification of the distribution of certain elements of the models allowing to consider a semi-parametric framework. In practice, the optimization problem defined in (13) can be solved using standard numerical methods such as, for example, the Broyden–Fletcher–Goldfarb–Shanno algorithm (see, e.g., Bonnans et al. 2006). Moreover, there exist closed-form expressions for the gradient of $\varvec{\nu }(\varvec{\gamma })$ with respect to $\varvec{\gamma }$ for a large class of models of practical interest, which allows to avoid numerical approximations of this quantity. The GMWM estimator is consistent and asymptotically normally distributed under arguably weak conditions (see Guerrier et al. 2013, 2021 for details). By the continuous mapping theorem and for any consistent estimator of $\textbf{x}_0$, say $\textbf{x}$, we have under technical requirements (see Guerrier et al. 2021 for details) that $\widehat{\varvec{\nu }}\left( \textbf{x}\right)$ is a consistent estimator of $\varvec{\nu }(\varvec{\gamma }_0)$. In particular, we propose to consider $\widetilde{\textbf{x}}$ as defined in (9) which satisfies

14 $$\begin{aligned} \widehat{\varvec{\nu }}\left( \widetilde{\textbf{x}}\right) \overset{p}{\longrightarrow }\ \varvec{\nu }(\varvec{\gamma }_0), \end{aligned}$$

and, therefore, under the conditions of Guerrier et al. (2013) we have

15 $$\begin{aligned} \widetilde{\varvec{\gamma }} = \widetilde{{\varvec{\gamma }}}\left( \widetilde{\textbf{x}}\right) \overset{p}{\longrightarrow } \varvec{\gamma }_0. \end{aligned}$$

Similarly to $\widetilde{\textbf{x}}$, the estimator $\widetilde{{\varvec{\gamma }}}$ is (asymptotically) less efficient than $\widehat{{\varvec{\gamma }}}$ as defined in (5). To narrow this gap, it is possible to consider instead the following procedure which iteratively recomputes $\widetilde{\textbf{x}}$ defined in (8) based on an updated estimator of $\varvec{\Sigma }({\varvec{\gamma }}_0)$. Starting at $j = 1$ with $\varvec{\Sigma }^{(0)} = \textbf{I}$, we define

16 $$\begin{aligned} \widetilde{\textbf{x}}^{(j)}= & {} \left\{ \textbf{A}^{\textrm{T}}\left( \varvec{\Sigma }^{(j-1)}\right) ^{-1} \textbf{A}\right\} ^{-1} \textbf{A}^{\textrm{T}}\left( \varvec{\Sigma }^{(j-1)}\right) ^{-1} \textbf{Y}, \nonumber \\ \widetilde{\varvec{\gamma }}^{(j)}= & {} \underset{\varvec{\gamma } \in \varvec{\Gamma }}{{{\,\textrm{argmin}\,}}}\; \left\{ \widehat{\varvec{\nu }}\left( \widetilde{\textbf{x}}^{(j)}\right) - \varvec{\nu }(\varvec{\gamma })\right\} ^{\textrm{T}}\varvec{\Omega } \left\{ \widehat{\varvec{\nu }}\left( \widetilde{\textbf{x}}^{(j)}\right) - \varvec{\nu }(\varvec{\gamma })\right\} ,\nonumber \\ \varvec{\Sigma }^{(j)}= & {} \varvec{\Sigma }\left( \widetilde{\varvec{\gamma }}^{(j)}\right) = {{\,\textrm{var}\,}}\left( \textbf{Y} | \widetilde{\varvec{\gamma }}^{(j)}\right) . \end{aligned}$$

This iterative procedure is illustrated in Fig. 1. In fact, we have that $\widetilde{\textbf{x}}^{(j)}$ is asymptotically efficient for all $j \geqslant 2$ in the sense that

17 $$\begin{aligned} \lim _{n \rightarrow \infty } \, {{\,\text {var}\,}}\left\{ \sqrt{a_n}\left( \widehat{{\textbf {x}}} {-} {\textbf {x}}_0 \right) \right\} {-} {{\, \text {var}\,}}\left\{ \sqrt{a_n} \left( \widetilde{{\textbf {x}}}^{(j)} {-} {\textbf {x}}_0 \right) \right\} {=} {\textbf {0}}. \end{aligned}$$

This result is a consequence of the consistency of $\widetilde{\varvec{\gamma }}^{(j)}$ for $j \geqslant 1$, the continuous mapping theorem and Slutsky’s theorem, provided that the function $\varvec{\Sigma }({\varvec{\gamma }})$ is continuous in ${\varvec{\gamma }}$. This is a plausible requirement which is satisfied for the majority of time series models. The derivation of Eq. (17) is detailed in Appendix A.2.

The procedure described in (16) is known as the *iterated* GMM, when iterated until convergence. The special case of $j = 2$ is the so-called two-step GMM widely used in econometrics (Greene 2003). In this article, our main focus is on providing a reliable yet computationally efficient estimator of $\varvec{\theta }_0$. For this reason, we opt for the convenient choices of $j \in \{1, \,2\}$ which corresponds to the following estimators:

18 $$\begin{aligned} \begin{aligned} \widetilde{{\varvec{\theta }}}_j&= \left[ \widetilde{\textbf{x}}^{(j) \mathrm T} \;\; \widetilde{\varvec{\gamma }}^{(j) \mathrm T} \right] ^{\textrm{T}}. \end{aligned} \end{aligned}$$

These particular choices are consistent with the statistical properties of this estimator, but they are based mainly on our empirical experience and desire for simplicity and is not necessarily an optimal choice.

Moreover, our approach can easily accommodate the presence of missing data. Indeed, the WV estimator $\widehat{\varvec{\nu }}\left( {\textbf{x}}\right)$ is a linear function of the wavelet coefficients, which are linear combinations of the observations. Thus, in the presence of missing observations, the WV can be computed on the *available* wavelet coefficients (i.e., the coefficients corresponding to non-missing observations) without impacting the expectation of $\widehat{\varvec{\nu }}\left( {\textbf{x}}\right)$ due to the linearity properties of the expectation operator.

The first estimator $\widetilde{{\varvec{\theta }}}_1$ is particularly computationally efficient, and its computational complexity is only of order $\mathcal {O}\{\log (n) n\}$, which remains unchanged when considering situations with or without missing data. Indeed, the computational complexity of the MLE is of order $\mathcal {O}(n^3)$ in most implementations. However, it can be reduced to $\mathcal {O}(n^2)$ under suitable conditions (see, e.g., Bos et al. 2013 and the references therein). The second $\widetilde{{\varvec{\theta }}}_2$ is slightly more computationally demanding but is asymptotically efficient for $\widetilde{\textbf{x}}^{(2)}$. Hereafter, we denote the estimator defined in Eq. (18) with one or two iterations as the GMWMX-1 and the GMWMX-2, respectively. The performances of the proposed estimators are illustrated and discussed in detail in Sect. 4.1. As previously mentioned, the iterative procedure described in (16), with $j > 2$, could be used to further improve the statistical properties of $\widetilde{{\varvec{\theta }}}_2$, but we do not pursue this direction here.

### Contributions

The general statistical framework proposed in the previous section has several advantages over the standard MLE. First, our approach is *semi-parametric* in the sense that the probability distribution $\mathcal {F}$ considered in (3) is left unspecified. Throughout this paper, we consider a general class for the probability distribution $\mathcal {F}$, which can be characterized by a set of mild regularity conditions. These advantageous features avoid the common assumption that the residuals $\varvec{\varepsilon }$ are issued from a multivariate normal distribution. Indeed, this assumption is often unrealistic in practice as the (estimated) residuals may have asymmetric and leptokurtic distributions. Consequently, our methodology offers statistical guarantees, such as consistency or asymptotic normality, without relying on strong parametric assumptions.

Secondly, the proposed approach is *computationally efficient* while preserving adequate statistical properties. The computational cost of our method is comparable to a single evaluation of the standard Gaussian likelihood function with its computational bottleneck corresponding to the inversion of an $n \times n$ matrix. Indeed, our two-step estimator $\widetilde{{\varvec{\theta }}}_2 = \left[ \widetilde{\textbf{x}}^{(2)\mathrm T} \;\; \widetilde{\varvec{\gamma }}^{(2) \mathrm T} \right]$ defined in (18) is consistent for $\varvec{\theta }_0$. Moreover, the estimator $\widetilde{\textbf{x}}^{(2)}$ for $\textbf{x}_0$ is *asymptotically efficient* and corresponds to the (asymptotically) *best unbiased estimator* in the sense of Hansen (2022). The estimator $\widehat{\varvec{\gamma }}$ for $\varvec{\gamma }_0$ has similar statistical properties to the ones of the MLE (at the Gaussian model) but possibly comes at the price of a marginally inflated variance due to the semi-parametric nature of the procedure. Moreover, a significant advantage of the proposed method is that its computational cost remains constant with the proportion of missing data.

Furthermore, our methodology is *scalable* as it provides a simple strategy using $\widetilde{{\varvec{\theta }}}_1$ defined in  (18) to marginally reduce the statistical properties of our estimator in order to considerably limit the computational burden. Indeed, in situations where large networks of GNSS stations are considered, the computational cost can be further reduced to become comparable to the computation of the standard least squares estimator. Consequently, large-scale problems can be solved within a few minutes on a standard computer.

## Results and discussions

### Simulation studies

In this section, we evaluate the performances of the GMWMX-1 and GMWMX-2 estimators defined in (18) as well as the validity of their associated confidence intervals compared to the MLE as implemented in the software Hector v1.9 of Bos et al. (2008) via Monte Carlo simulations. We consider a simulated scenario based on (2) for the functional model. As for the stochastic model, we first consider $\varvec{\varepsilon }$ to be the sum of a power law and a Gaussian white noise, which is a widely accepted model (Zhang et al. 1997; Bos et al. 2008; Klos et al. 2014). The values of the functional parameters are fixed as follows: $a = 0$, $b = 5$ mm/year, and the annual periodic motion has an amplitude of 2.5 mm. Moreover, we consider a model where three offsets are present and known, i.e., $n_g = 3$. For the stochastic part, we consider $\sigma ^2_\text {PL} = 10$ mm/year and $d = 0.4$, while the variance of the white noise is $\sigma ^2_\text {WN} = 15$ mm$^2$. We fix the functional and stochastic parameters by considering values at the center of the distribution of the estimated parameters for the Z-axis of the stations considered in the case study (see Sect. 4.2). All our simulations are based on $B = 10^3$ Monte Carlo replications.

**Fig. 2:**
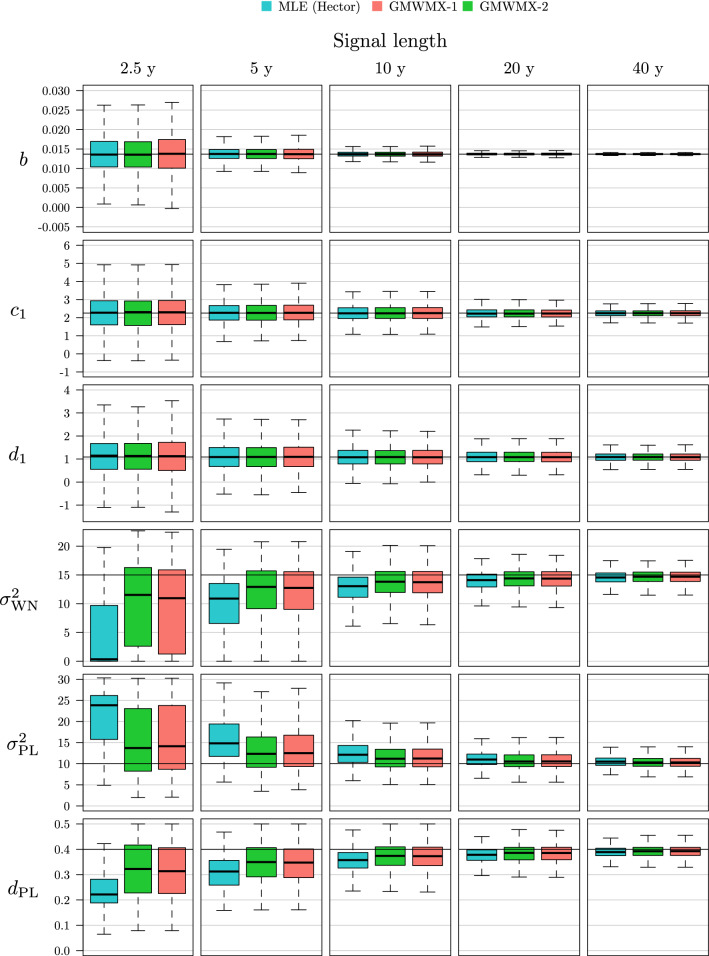
Boxplots of the estimated deterministic and stochastic parameters with method GMWMX-1, GMWMX-2 and the MLE for different sample sizes for the simulation considering a white noise coupled with a power law process as the stochastic model denoted as Setting A1. The black line indicates the true value of the estimated parameter

We first compare the performance of the GMWMX-1, the GMWMX-2 and the MLE by considering different lengths of GNSS daily position time series, i.e., 2.5, 5, 10, 20 and 40 years. We consider 10% of missing observations for each simulated signal which corresponds approximately to the estimated median number of missing data of publicly available datasets (see Bos et al. 2013 for more details). For the stochastic model defined above, we denote this simulation setting as Setting A1. The estimated parameters from the (geophysical) functional model and for the stochastic one for different lengths of signal are represented in Fig. 2. It can be observed how the functional parameters are estimated well by the three methods, although the GMWMX-1 exhibits a slightly increased variance for the trend parameter *b*. Regarding the stochastic parameters, all methods tend to estimate a higher variance for the power law noise at the expense of the white noise. However, this bias appears slightly larger for MLE and the GMWMX-based methods seem to provide a small gain in accuracy for these parameters, an observation which echoes with the empirical results of Guerrier et al. (2013).

**Fig. 3:**
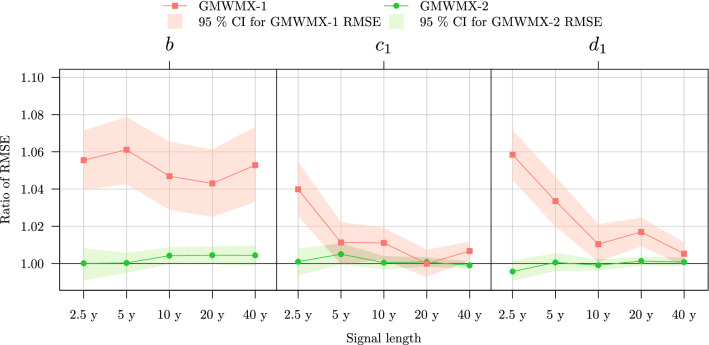
Ratio of the estimated RMSE of the GMWMX-1 and GMWM-2 compared to the MLE for the functional parameters *b*, $c_1$ and $d_1$ as a function of the sample size for the simulation considering a white noise coupled with a power law process as the stochastic model denoted as Setting A1

In order to assess the performance of the methods more thoroughly, we consider the ratio of the root-mean-square error (RMSE) of GMWMX-1 and GMWMX-2 over the one of the MLE. The GMWMX-2 is expected to have very similar finite sample performances as the MLE for the functional parameters due to their asymptotic equivalence presented in (17). The results are presented in Fig. 3 for some of the functional parameters (i.e., *b*, $c_1$ and $d_1$). As expected, the GMWMX-2 appears to have an identical RMSE compared to the MLE. Indeed, the ratio of the RMSEs is non-distinguishable from 1 when accounting for the simulation error (obtained by nonparametric bootstrap). However, these methods provide better results than the GMWMX-1, but the improvement is relatively small ranging from 0% to 6%. These results are in line with the theoretical properties of the estimators presented in Sect. 3.1.

**Fig. 4:**
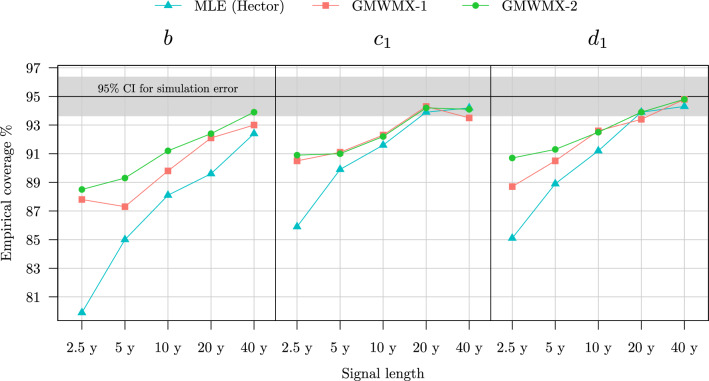
Empirical coverage of the confidence intervals at level $1 - \alpha = 0.95$ for the functional parameters *b*, $c_1$ and $d_1$ for GMWMX-1, GMWMX-2 and the MLE as a function of the sample size for the simulation considering a white noise coupled with a power law process as the stochastic model denoted as Setting A1. The grey area represents a 95% confidence intervals of the simulation errors based on $10^3$ Monte Carlo replicates

Another significant result is related to the validity of the confidence intervals that can be constructed for the functional parameters. The estimated uncertainty for each parameter allows to construct confidence intervals at any chosen confidence level, i.e., the intervals within which the true parameter should lie with the given probability. As a rule of thumb, if ${\tilde{\sigma }}$ is the estimated uncertainty for a given parameter which is asymptotically normally distributed, then the interval constructed around the estimated value $\pm 1.96 {\tilde{\sigma }}$ yields the approximately 95% confidence interval for that parameter (see Appendix F for details). With Monte Carlo simulations, the true parameter values are known: this makes it possible to verify the validity of the constructed confidence intervals, i.e., if they include the true parameter value with the required probability. The empirical coverage of the deterministic parameters, defined as the proportion of simulations in which the true value of the parameters is inside the computed confidence intervals, is shown for the MLE, the GMWMX-1 and the GMWMX-2 in Fig. 4. We observe that all methods yield empirical coverages close to the chosen confidence level of 95%. Therefore, the uncertainty for the functional parameters is reasonably estimated by all methods. However, the GMWMX-2 (and to a lesser degree the GMWMX-1) appears to present empirical coverages that are closer to the chosen confidence level of 95%. In particular, the GMWMX-2 provides more accurate confidence intervals for the trend parameter *b* for all sample sizes with respect to both the GMWMX-1 and the MLE in the considered case. These results may be explained by the smaller bias of the GMWMX-based methods for the stochastic parameters (as shown in Fig. 2) compared to the MLE.

**Fig. 5:**
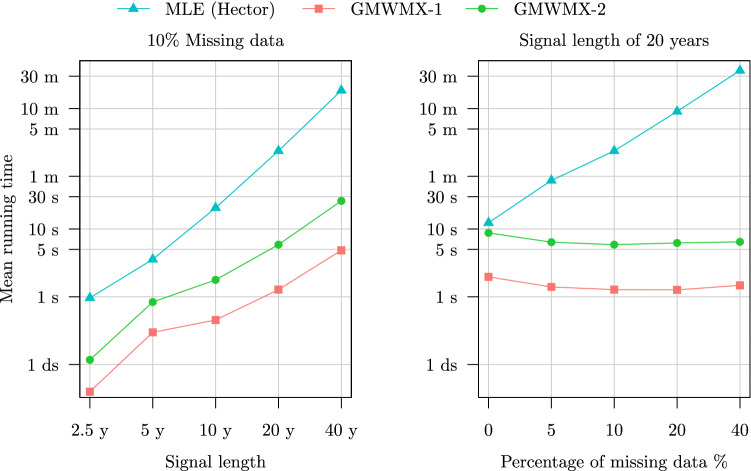
Mean running time of the MLE, the GMWMX-1 and the GMWMX-2 as a function of the sample size and of the proportion of missing observations for the simulation considering a white noise coupled with a power law process as the stochastic model denoted as Setting A1 and Setting A2

An important advantage of the proposed method is its computational efficiency and significantly shorter running time compared to the likelihood-based methods. In the left panel of Fig. 5, we present the running time of the MLE and GMWMX estimators as a function of the sample size *n* for Setting A1. While Hector takes on average 1 s for the smallest sample size of 2.5 years, corresponding to 912 data points and up to 18 min for the largest sample size of 40 years, the GMWMX-1 takes on average less than 5 s for the largest sample size considered of 40 years. Therefore, the GMWMX-1 is between 20 and 200 times faster than the MLE in the cases considered in this simulation. Regarding the GMWMX-2, the increased statistical efficiency (i.e., lower asymptotic variance) comes at the price of a longer running time because of the need to compute the inverse of ${\varvec{\Sigma }}(\tilde{{\varvec{\gamma }}}^{(1)})$ once. However, in this setting, the GMWMX-2 is still between 10 and 40 times faster than the MLE while providing statistically equivalent results as shown in Fig. 3.

Next, we investigate the impact of the proportion of missing observations as well as the stochastic model on the statistical performance and computation time of the considered methods. First, we replicate the previous simulation with a sample size of 20 years but considering a proportion of missing data varying from 0 to 40%. We refer to this simulation scenario as Setting A2. Then, we replicate the first two simulations with the same functional model but considering a stochastic model composed of a white noise coupled with a Matérn process with parameters: $\sigma ^2_\text {WN}=5$ mm$^2$, $\sigma ^2_\text {MAT}=25$ mm/year, $\lambda _\text {MAT}=0.1$ and $\alpha _\text {MAT}=1.1$. Thus, we consider (i) 10% of missing observations and sample size varying from 2.5 to 40 years and (ii) a sample size corresponding to 20 years and a proportion of missing data varying from 0 to 40% with this new stochastic model. We, respectively, denote these two additional simulation settings as Setting B1 and Setting B2. The results for Setting A2 are presented in Appendix C, while results for Settings B1 and B2 are presented, respectively, in Appendices D and E. Essentially, the conclusions that can be drawn for Settings A2, B1 and B2 in terms of statistical properties of the considered estimator are similar to our first simulation with Setting A1. Indeed, it can be observed in Figs. 9, 11 and 13 that the functional parameters are similarly estimated with the MLE and the GMWMX-2, while the GMWMX-1 has a slightly increased variance. Regarding the stochastic parameters, all methods are comparable and in some cases a small gain in accuracy can be observed for GMWMX-based methods (see, e.g., Figs. 9 and 11). Similarly to our previous simulation, all methods provide similar performances in terms of empirical coverage (see Figs. 10, 12 and 14 for more details). In some instances, GMWMX-2 appears to provide slightly more accurate confidence intervals compared to both the GMWMX-1 and the MLE (see, e.g., Fig. 12).

**Fig. 6:**
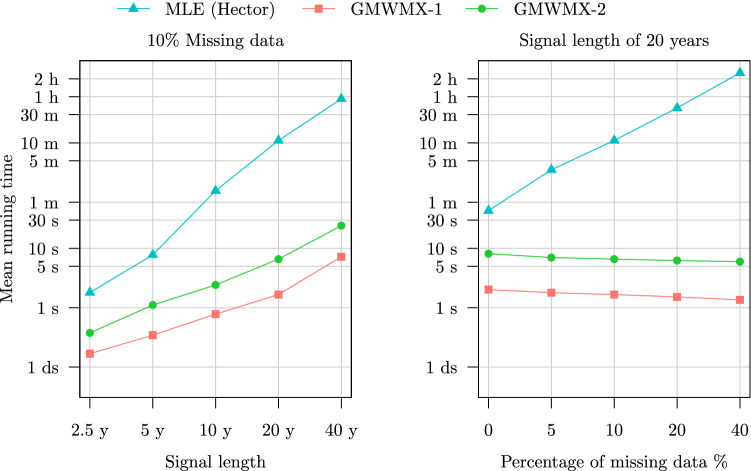
Mean running time of the MLE, the GMWMX-1 and the GMWMX-2 as a function of the sample size and of the percentage of missing observations for the simulation considering a white noise coupled with a Matérn process as the stochastic model denoted as Setting B1 and Setting B2

In terms of computational time, it can be observed in the right panel of Fig. 5 and of Fig. 6 that the computation cost of the GMWMX estimators appears to be invariant to the proportion of missing observations, while this is not the case for the MLE. More precisely, in Setting A2 presented in the right panel of Fig. 5, we observe that while the MLE takes on average just above 12 s to estimate a time series of 20 years without any missing observations, over 36 min are needed on average when considering a large missing values proportion of 40%. On the contrary, the GMWMX-1 takes on average less than 2 s, while the GMWMX-2 takes about 7 s on average to estimate the parameters of a time series of 20 years, regardless of the proportion of missing observations. In Setting B1 presented in the left panel of Fig. 6, the MLE takes up to 55 min on average to estimate a signal of 20 years with 10% missing observations, while the GMWMX-1 takes less than 8 s and the GMWMX-2 presents an average computation time of 24 s to estimate the same signal. In Setting B2 presented in the right panel of Fig. 6, it can be noted that the MLE takes more than 2.5 hours in the case of 40% of missing observations, while the GMWMX-1 has an average computation time of less than 2 s and the GMWMX-2 has an average computation time of less than 8 s. Hence, depending on the stochastic model considered and the percentage of missing observations, the GMWMX-1 is between 10 to more than 6600 times faster than the MLE.

Based on these empirical results, three takeaways can be drawn regarding the computational performance of the GMWMX estimators over the MLE. First, we note that when fixing the proportion of missing observations and increasing the sample size, the computational time of the MLE seems to increase faster than the ones of the GMWMX methods. Second, when considering a fixed sample size and increasing the proportion of missing observations, the computational time of the GMWMX methods appears to be invariant with respect to the percentage of missing data, while this is not the case for the MLE. Finally, we observe that the computational time of the MLE can increase noticeably depending on the stochastic model considered, while it is not the case for the GMWMX methods, hence leading to a notable difference in execution time in the presence of missing observations in the signal.

We would like to highlight that, due to the diversity of the factors involved in the runtime of the evaluation of a software instruction (i.e., hardware of the installation, programming language in which the executed instruction is implemented, potential parallel implementation of the code, level of code optimization of the methods compared, etc.), it is difficult to present a fair and exhaustive comparison of performances when comparing execution times. As Hector and the GMWMX methods could be implemented for parallel computation in a different fashion and executed on different processing units, our results report the computation time for a single thread execution. The simulations were executed on the high-performance computing cluster of the University of Geneva. Moreover, for each realization of a simulation setting, the different methods compared were run on the same CPU models (either Intel Xeon Gold 6244, Intel Xeon Gold 6240 or AMD EPYC-7742) to ensure a fair comparison.

Further simulation studies suggest that the GMWMX-2 yields confidence intervals with marginally better empirical coverage with respect to the MLE or the GMWMX-1 when the residuals $\varvec{\varepsilon }$ do not follow a multivariate Gaussian distribution (e.g., skewed Student’s t-distribution). However, the inferential advantages of the proposed semi-parametric method outside of the Gaussian model are beyond the scope of our study and are left for further research.

### Case study

We apply our method to daily GNSS coordinate time series. We use measurements from 33 continuously operating GNSS receivers distributed over the east coast of the USA. The daily position time-series result from the processing released by the Pacific Northwest Geodetic Array at the Central Washington University (PANGA/CWU, Herring et al. 2016; He et al. 2021) computed within the International Terrestrial Reference Frame 2014 (Altamimi et al. 2016).

**Fig. 7:**
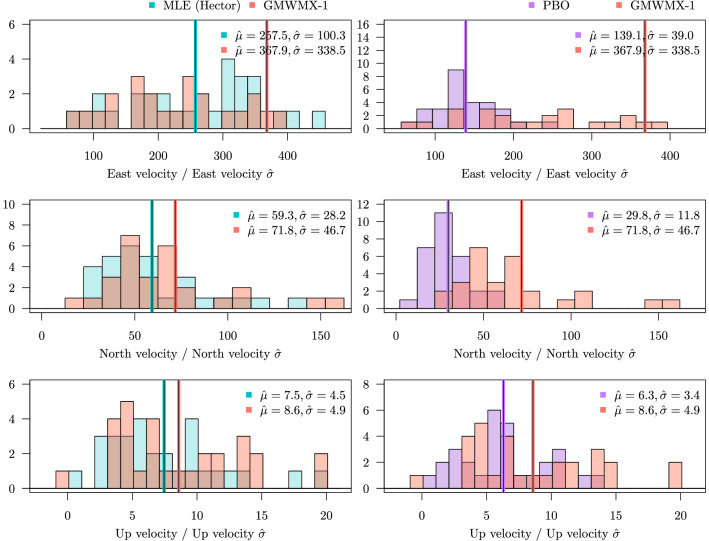
Ratios between estimated North-East velocities and crustal uplift divided by their respective estimated standard deviation for the GMWMX-1, the MLE and the PBO product for 33 GNSS receivers distributed over the East coast of the USA

**Fig. 8:**
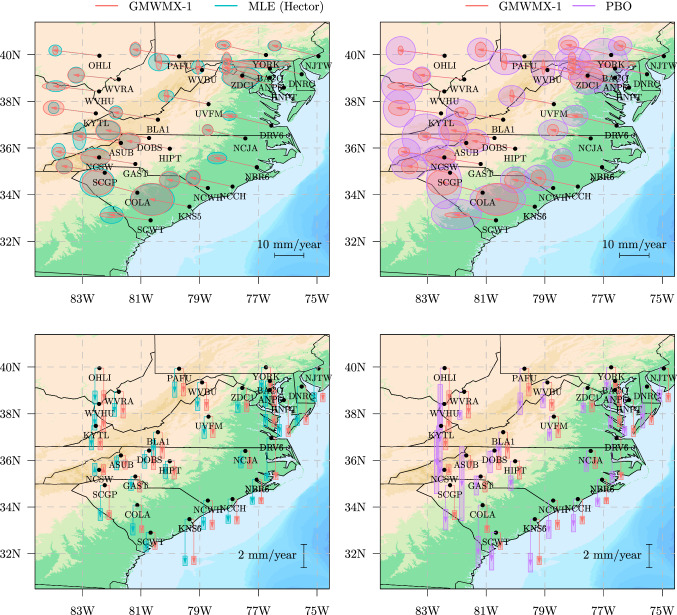
Estimated North-East velocity solutions and crustal uplift for 33 GNSS receivers distributed over the East coast of the USA using i) the GMWMX-1, ii) Hector software (MLE), iii) the PBO product

The analysis center PANGA/CWU computes the daily positions using the precise point positioning method with the GIPSY software developed by NASA’s Jet Propulsion Laboratory (JPL). The latter also provides the necessary satellite ephemerides, clock corrections, and wide-lane phase bias estimates (Herring et al. 2016). The station positions were loosely constrained during the initial estimation and subsequently transformed into the International Terrestrial Reference Frame (ITRF2014) using only the translation and rotation (Altamimi et al. 2016), but not scale, components of the JPL-provided Helmert transformations. Readers interested in the comprehensive discussion on the choice of the processing parameters can refer to Herring et al. (2016) and He et al. (2021).

We use the resulting daily position solution time series to estimate the tectonic rate and the associated uncertainties with the GMWMX-1 and the MLE, as implemented in the Hector software (Bos et al. 2008). For comparison purposes, we have also included the velocity solutions provided by the PBO center (Herring et al. 2016). The 33 GNSS stations have at least 8 years of continuous observations (see Fig. 8). The same time range is carefully selected for each station in order to do a genuine comparison between the estimated tectonic rate with Hector, GMWMX-1 and the PBO solutions. The input data contain outliers. We employ the utility removeoutliers included in the Hector package since outlier rejection is beyond the scope of the current work.

For both GMWMX-1 and the MLE, we chose the functional model presented in (2), which includes a seasonal and half-seasonal component and multiple offsets. The offset times ($t_k$) are provided by PBO, while we estimate the amplitudes ($g_k$). The stochastic model is a sum of a power law and a white noise, also used to perform the simulations presented in the previous section.

To quantify the difference between the solutions from a statistical perspective, Fig. 7 displays the range of rates and uncertainties, i.e., the ratio between the estimates and the associated uncertainties. Our solution (GMWMX-1) and the estimates with Hector (MLE) compare well within error. In terms of mean value, the ratio difference is $~6 \%$ (East), $~9.7 \%$ (North) and $~9.5 \%$ (Up). For the East component, the ratio is much higher than the other ones, suggesting that the uncertainty is small compared to the tectonic rate. Note that the Up component is known to contain 3 times more noise than the horizontal coordinates (Montillet et al. 2020). Correspondingly, the uncertainty is large resulting in a small ratio. Looking at the ratio difference between the estimates released by PBO and GMWMX-1, the results are $~43 \%$ (East), $~48 \%$ (North) and $~14 \%$ (Up). This large difference is basically due to the uncertainty associated with the tectonic rate. Appendix G displays the results for GMWMX-2 which are very similar to the ones for GMWMX-1. Given that each GNSS station records observations for the three coordinates (East, North, Up) and that the mean size of each time series is approximately 10 years, ranging from 8 to 15 years, the computing time for the GMWMX-1 for the whole GNSS network is below 40 s, while in comparison, Hector’s processing time is approximately 23 min.

Figure 8 displays the various solutions (i.e., GMWMX-1, Hector and PBO product). Note that we have separated the arrows on the maps of the crustal uplift for the sake of clarity. The values are shown in Tables 1, 2 and 3 in Appendix G. Overall, the solutions agree with the results published by Perosanz (2019) and Métivier et al. (2020). The good agreement between Hector and GMWMX-1 can be seen visually for the East, North, and Up components. They validate the results from the simulated time series and show good agreement with Hector processing. The PBO solution is in line with the MLE and GMWMX-1 results for the amplitude of the tectonic rate and the crustal uplift. However, the uncertainties with this product are generally larger which is due to the difference between the methods. The GMWMX-1 and Hector are both jointly estimating a stochastic noise together with a geophysical model, whereas the PBO solution is based on a fast statistical approach. The method relies on a Kalman filter based on a first-order Gauss–Markov noise characteristic without any further analysis on the noise structure of the data (Floyd and Herring 2020). The difference in the uncertainties is emphasized by the crustal uplift values. This result explains the ratio difference between the PBO solutions and the other methods in Fig. 7.

## Conclusions

In this contribution, we propose a new method called the GMWMX to estimate the parameters of linear models with correlated residuals, which we apply to the analysis of GNSS daily position time series. The GMWMX allows a computationally efficient estimation of stochastic and functional (geophysical) models. Moreover, our approach is semi-parametric in the sense that the underlying distribution is left unspecified. Unlike the MLE, the GMWMX remains consistent and asymptotically normally distributed for all zero-mean probability distributions satisfying mild regularity conditions. Our approach is scalable in the sense that two estimators (GMWMX-1 and GMWMX-2) are proposed. The first estimator GMWMX-1 is particularly computationally efficient and presents a reduction in computational time ranging from 10 to a few thousand times faster compared to the MLE depending on the considered stochastic model, the length of the time series and the amount of missing observations. However, this estimator comes at the price of marginally deteriorated statistical properties. The second estimator GMWMX-2 has an increased processing time than the GMWMX-1 but remains considerably faster than the MLE with a computation time between 4 and over 1500 times smaller than the MLE depending on the considered stochastic model, the sample size and the percentage of missing observations. The GMWMX-2 is shown to be asymptotically efficient (and therefore asymptotically equivalent to the MLE) for the linear functional parameters. Moreover, this estimator corresponds to the (asymptotically) best unbiased estimator in the sense of Hansen (2022). Both GMWM-based estimators are consistent and asymptotically normally distributed under arguably weak conditions (see Guerrier et al. 2013, 2021 for details).

Our theoretical findings are validated considering different simulated scenarios which consider different stochastic models, different sample sizes, and various proportions of missing observations. Our results indicate that the GMWMX-1 is 10 to more than 6600 times faster than the MLE but comes at the price of a marginally inflated RMSE (around 5% on average) compared to the MLE for the functional parameters. The GMWMX-2 is 4–1500 times faster than the MLE, but its statistical performance is indistinguishable from the MLE for the functional parameters (less than 0.01% difference in terms of RMSE). Both the GMWMX-1 and the GMWMX-2 lead to comparable results to the MLE for the estimation of the stochastic parameters.

In order to support the simulation studies, we apply our algorithm to the analysis of real observations recorded from a network of 33 GNSS stations located in the eastern part of the USA. These selected stations have registered at least 8 years of data. Our results indicate that the use of the GMWMX-1 gives comparable results to the MLE with a widely assumed stochastic model (a white noise summed with a power law process). Overall, the results are nearly identical (with a difference of less than $2 \%$) between the MLE and GMWMX-1 when looking at the estimated tectonic rate and crustal uplift at each station. The clear advantage of the GMWMX-1 is the processing time which is approximately 40 times lower than the one of the MLE with a marginal difference in terms of RMSE. Similar velocity estimates are obtained for the MLE and the GMWMX as well as for the stochastic parameters, highlighting the consistency of the two estimators. However, the associated uncertainties can vary up to $90 \%$ compared to the PBO solution. This large variation can be explained by the fast statistical approach used for the PBO solution which is based on an approximated stochastic noise model.

The GMWMX allows to jointly estimate a functional and a stochastic noise model and produces accurately reliable uncertainties of the estimated parameters. It is a computationally efficient and scalable estimator based on simple statistical concepts and will be ideal to process large-scale networks which include thousands of GNSS stations.

## Figures and Tables

**Fig. 9:**
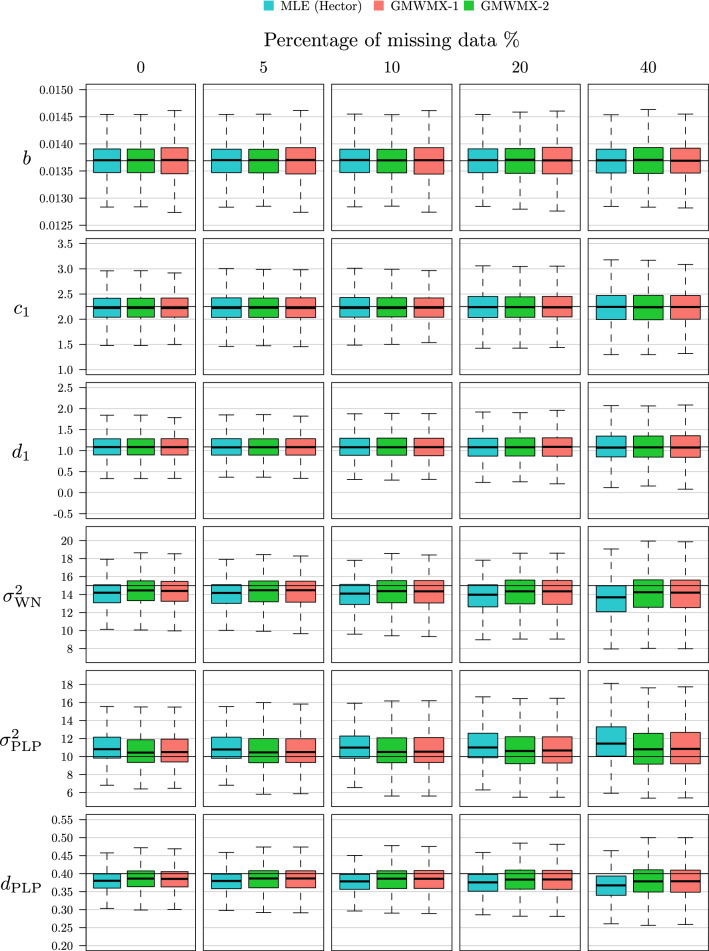
Boxplots of the estimated deterministic and stochastic parameters with method GMWMX-1 and the MLE for different percentages of missing observations for the simulation considering a white noise coupled with a power law process as the stochastic model denoted as Setting A2. The black line indicates the true value of the estimated parameter

**Fig. 10:**
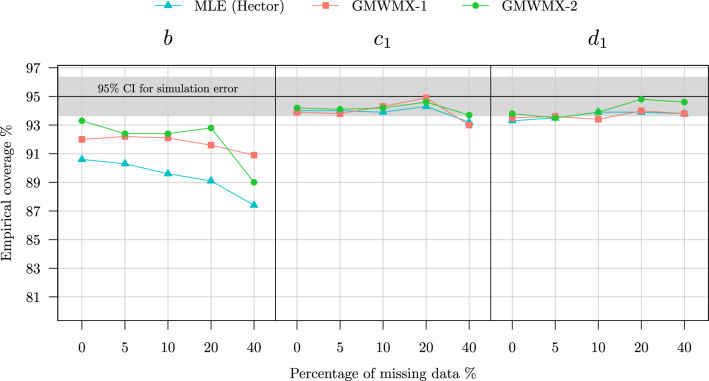
Empirical coverage of the confidence intervals at level $1 - \alpha = 0.95$ for the functional parameters *b*, $c_1$ and $d_1$ for GMWMX-1, GMWMX-2 and the MLE as a function of the percentage of missing observations for the simulation considering a white noise coupled with a power law process as the stochastic model denoted as Setting A2. The gray area represents a 95% confidence interval of the simulation errors based on $10^3$ Monte Carlo replicates

**Fig. 11:**
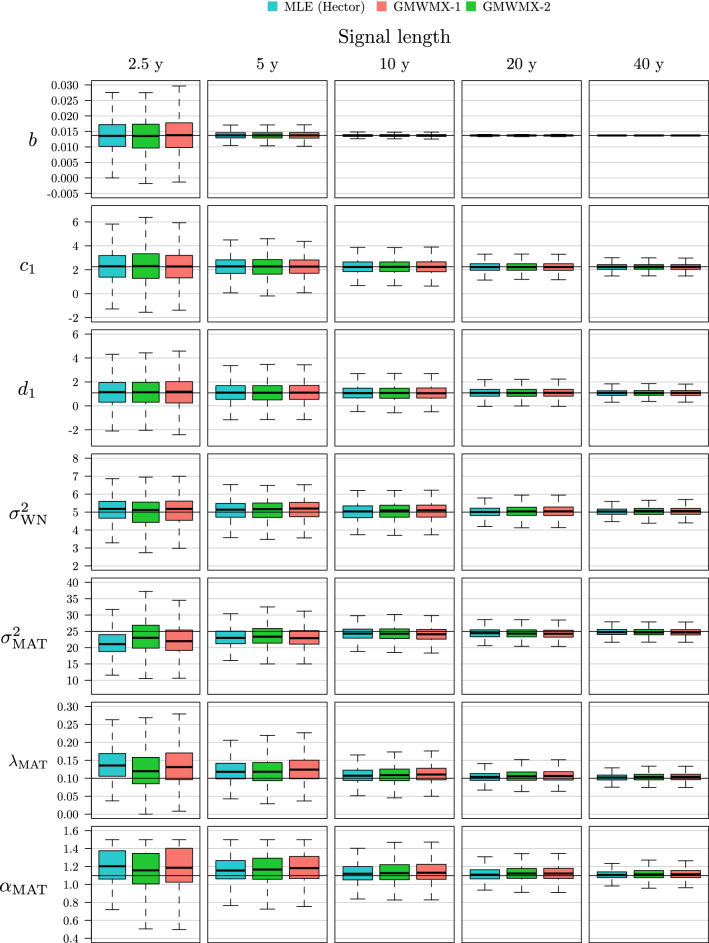
Boxplots of the estimated deterministic and stochastic parameters with method GMWMX-1, GMWMX-2 and the MLE for different sample sizes for the simulation considering a white noise coupled with a Matérn process as the stochastic model denoted as Setting B1. The black line indicates the true value of the estimated parameter

**Fig. 12:**
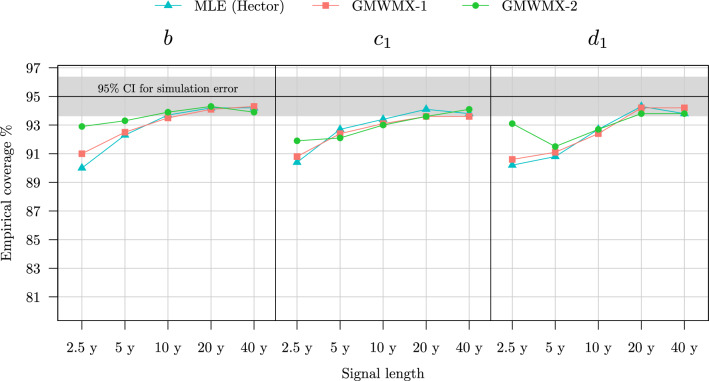
Empirical coverage of the confidence intervals at level $1 - \alpha = 0.95$ for the functional parameters *b*, $c_1$ and $d_1$ for GMWMX-1, GMWMX-2 and the MLE as a function of sample size for the simulation considering a white noise coupled with a Matérn process as the stochastic model denoted as Setting B1. The gray area represents a 95% confidence interval of the simulation errors based on $10^3$ Monte Carlo replicates

**Fig. 13:**
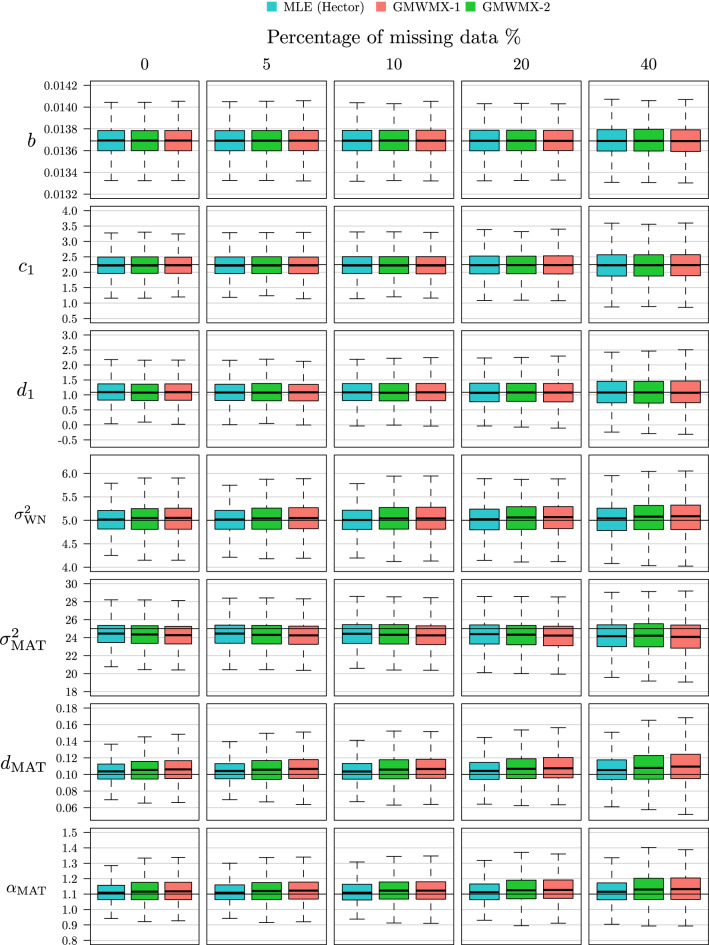
Boxplots of the estimated deterministic and stochastic parameters with method GMWMX-1, GMWMX-2 and the MLE for different percentages of missing observations for the simulation considering a white noise coupled with a Matérn process as the stochastic model denoted as Setting B2. The black line indicates the true value of the estimated parameter

**Fig. 14:**
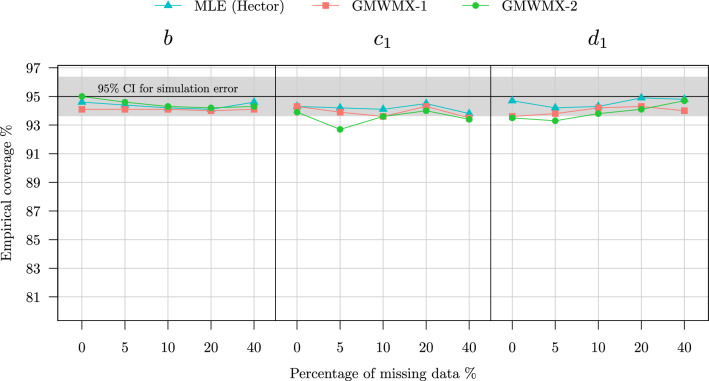
Empirical coverage of the confidence intervals at level $1 - \alpha = 0.95$ for the functional parameters *b*, $c_1$ and $d_1$ for GMWMX-1, GMWMX-2 and the MLE as a function of the percentage of missing observations for the simulation considering a white noise coupled with a Matérn process as the stochastic model denoted as Setting B2. The gray area represents a 95% confidence interval of the simulation errors based on $10^3$ Monte Carlo replicates

**Fig. 15:**
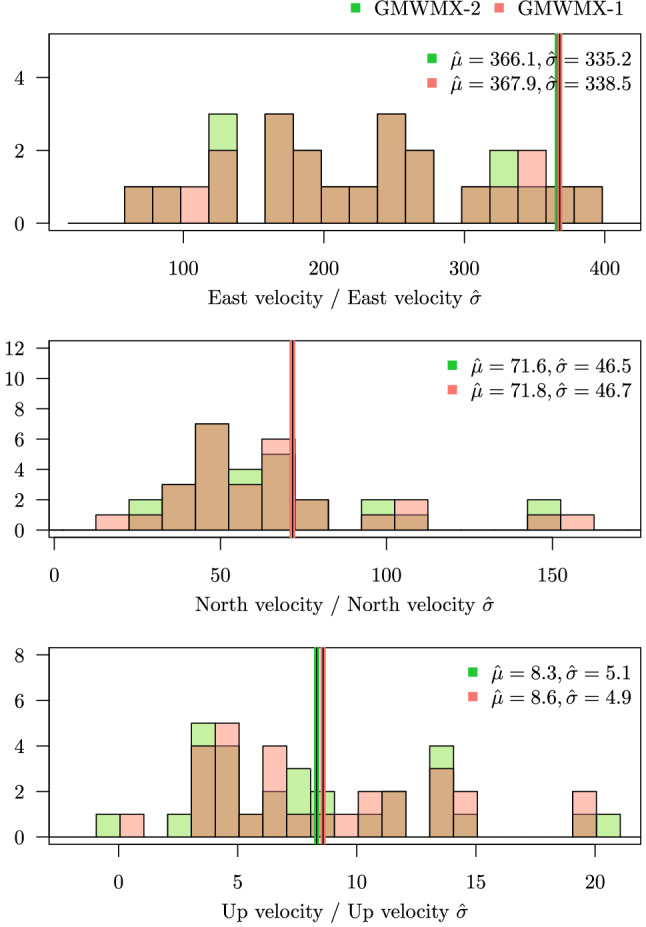
Ratios between estimated North-East velocities and crustal uplift divided by their respective estimated standard deviation for the GMWMX-2 and the GMWMX-1 for 33 GNSS receivers distributed over the east coast of the USA

## A Mathematical derivation

### A.1 Derivation of Eq. (11)

By comparing (10) with (6), we have

$$\begin{aligned} \lim _{n \rightarrow \infty } \;&{{\,\textrm{var}\,}}\left\{ \sqrt{a_n} \left( \widetilde{\varvec{\textbf{x}}} - \varvec{\textbf{x}}_0 \right) \right\} - {{\,\textrm{var}\,}}\left\{ \sqrt{a_n} \left( \widehat{\varvec{\textbf{x}}} - \varvec{\textbf{x}}_0 \right) \right\} \\&=a_n\left( \textbf{A}^{\textrm{T}}\textbf{A} \right) ^{-1} \textbf{A}^{\textrm{T}}\varvec{\Sigma }(\varvec{\gamma }_0) \textbf{A} \left( \textbf{A}^{\textrm{T}}\textbf{A} \right) ^{-1} \\ {}&\qquad - a_n\left( \textbf{A}^{\textrm{T}}\varvec{\Sigma }(\varvec{\gamma }_0)^{-1} \textbf{A} \right) ^{-1} \\&= a_n \left( \textbf{A}^{\textrm{T}}\textbf{A} \right) ^{-1} \textbf{A}^{\textrm{T}}\varvec{\Sigma } (\varvec{\gamma }_0)^{1/2} \\&\Bigg \{\textbf{I}- \varvec{\Sigma }(\varvec{\gamma }_0)^{-1/2} \textbf{A}\left( \textbf{A}^{\textrm{T}}\varvec{\Sigma }(\varvec{\gamma }_0)^{-1} \textbf{A} \right) ^{-1} \textbf{A}^{\textrm{T}}\varvec{\Sigma }(\varvec{\gamma }_0)^{-1/2}\Bigg \}\\&\varvec{\Sigma }(\varvec{\gamma }_0)^{1/2} \textbf{A} \left( \textbf{A}^{\textrm{T}}\textbf{A} \right) ^{-1}. \end{aligned}$$

Then, by defining $\textbf{B}= \varvec{\Sigma }(\varvec{\gamma }_0)^{-1/2} \textbf{A}\left( \textbf{A}^{\textrm{T}}\varvec{\Sigma }(\varvec{\gamma }_0)^{-1} \textbf{A} \right) ^{-1} \textbf{A}^{\textrm{T}}\varvec{\Sigma }(\varvec{\gamma }_0)^{-1/2}$ and noticing that $\textbf{B}$ is idempotent, we obtain

$$\begin{aligned}&a_n \left( \textbf{A}^{\textrm{T}}\textbf{A} \right) ^{-1} \textbf{A}^{\textrm{T}}\varvec{\Sigma }(\varvec{\gamma }_0)^{1/2}\\&\quad \left\{ \textbf{I}- \varvec{\Sigma }(\varvec{\gamma }_0)^{-1/2} \textbf{A}\left( \textbf{A}^{\textrm{T}}\varvec{\Sigma }(\varvec{\gamma }_0)^{-1} \textbf{A} \right) ^{-1} \textbf{A}^{\textrm{T}}\varvec{\Sigma }(\varvec{\gamma }_0)^{-1/2}\right\} \\ {}&\varvec{\Sigma }(\varvec{\gamma }_0)^{1/2} \textbf{A} \left( \textbf{A}^{\textrm{T}}\textbf{A} \right) ^{-1}\\&\quad = a_n \left( \textbf{A}^{\textrm{T}}\textbf{A} \right) ^{-1} \textbf{A}^{\textrm{T}}\varvec{\Sigma }(\varvec{\gamma }_0)^{1/2} \\&\quad \left( \textbf{I}-\textbf{B}\right) \left( \textbf{I}-\textbf{B}\right) ^{\textrm{T}}\varvec{\Sigma }(\varvec{\gamma }_0)^{1/2} \textbf{A} \left( \textbf{A}^{\textrm{T}}\textbf{A} \right) ^{-1}\\&\quad = a_n \left\{ \left( \textbf{A}^{\textrm{T}}\textbf{A} \right) ^{-1} \textbf{A}^{\textrm{T}}\varvec{\Sigma }(\varvec{\gamma }_0)^{1/2} \left( \textbf{I}-\textbf{B}\right) \right\} \\&\quad \left\{ \left( \textbf{A}^{\textrm{T}}\textbf{A} \right) ^{-1} \textbf{A}^{\textrm{T}}\varvec{\Sigma }(\varvec{\gamma }_0)^{1/2} \left( \textbf{I}-\textbf{B}\right) \right\} ^{\textrm{T}}. \end{aligned}$$

Thus, we obtain

$$\begin{aligned} \lim _{n \rightarrow \infty } \; {{\,\textrm{var}\,}}\left\{ \sqrt{a_n} \left( \widetilde{\varvec{\textbf{x}}} - \varvec{\textbf{x}}_0 \right) \right\} - {{\,\textrm{var}\,}}\left\{ \sqrt{a_n} \left( \widehat{\varvec{\textbf{x}}} - \varvec{\textbf{x}}_0 \right) \right\} > \textbf{0}, \end{aligned}$$

for .

### A.2 Derivation of Eq. (17)

Fix any $j \geqslant 2$. To compare our estimator to the MLE, we assume $\textbf{Y} \sim \mathcal {N}\{\textbf{A} \textbf{x}_0, \varvec{\Sigma }(\varvec{\gamma }_0)\}$. Thus, we have that $\widetilde{\textbf{x}}^{(j)}$ conditionally on $\widetilde{\varvec{\gamma }}^{(j-1)}$ is normally distributed, i.e.,

A.1 $$\begin{aligned}{} & {} \sqrt{a_n} \left( \widetilde{\textbf{x}}^{(j)} - \textbf{x}_0 \right) \Big |\, \widetilde{\varvec{\gamma }}^{(j-1)} \sim \mathcal {N} \left\{ \textbf{0},\textbf{H}_n \big (\widetilde{\varvec{\gamma }}^{(j-1)}\big )\right\} , \end{aligned}$$

where

$$\begin{aligned} \begin{aligned} \textbf{H}_n \big ({\varvec{\gamma }}\big )&= {{\,\textrm{var}\,}}\left\{ \sqrt{a_n} \left( \widetilde{\textbf{x}}^{(j)} - \textbf{x}_0 \right) \Big | \, \varvec{\gamma }\right\} \\&= a_n \left\{ \textbf{A}^{\textrm{T}}\varvec{\Sigma }^{-1}(\varvec{\gamma }) \textbf{A} \right\} ^{-1}\\&\textbf{A}^{\textrm{T}}\varvec{\Sigma }^{-1}(\varvec{\gamma }) \varvec{\Sigma }(\varvec{\gamma }_0) \varvec{\Sigma }^{-1}(\varvec{\gamma }) \textbf{A} \left\{ \textbf{A}^{\textrm{T}}\varvec{\Sigma }^{-1}(\varvec{\gamma }) \textbf{A} \right\} ^{-1} . \end{aligned} \end{aligned}$$

By the continuity of matrix inversion and assuming the function $\varvec{\Sigma }(\varvec{\gamma })$ to be continuous in $\varvec{\gamma }$, the function $\textbf{H}_n \big ({\varvec{\gamma }}\big )$ is continuous in $\varvec{\gamma }$. With the same arguments used to obtain Eq. (15), we have $\widetilde{\varvec{\gamma }}^{(j-1)} = {\varvec{\gamma }}_0 + o_p(1)$. Then, by the continuous mapping theorem we get $\textbf{H}_n \big (\widetilde{\varvec{\gamma }}^{(j-1)}\big ) = \textbf{H}_n \big ({\varvec{\gamma }}_0\big ) + o_p(1)$. Therefore, by the law of total variance and Eq. (A.1), we can derive that

$$\begin{aligned} \begin{aligned} {{\,\textrm{var}\,}}\left\{ \sqrt{a_n} \left( \widetilde{\textbf{x}}^{(j)} - \textbf{x}_0 \right) \right\}&\!=\! \mathbb {E} \left[ {{\,\textrm{var}\,}}\!\left\{ \sqrt{a_n} \!\left( \widetilde{\textbf{x}}^{(j)} \!-\! \textbf{x}_0 \right) \!\Big | \, \widetilde{\varvec{\gamma }}^{(j\!-\!1)}\right\} \right] \\&\qquad + {{\,\textrm{var}\,}}\!\left[ \mathbb {E} \!\left\{ \sqrt{a_n} \!\left( \widetilde{\textbf{x}}^{(j)} \!-\! \textbf{x}_0 \right) \! \Big | \, \widetilde{\varvec{\gamma }}^{(j-1)}\right\} \right] \\&= \mathbb {E} \left[ {{\,\textrm{var}\,}}\!\left\{ \sqrt{a_n}\! \left( \widetilde{\textbf{x}}^{(j)} \!-\! \textbf{x}_0 \right) \!\Big |\! \, \widetilde{\varvec{\gamma }}^{(j\!-\!1)}\right\} \right] \\&= \mathbb {E}\left[ \textbf{H}_n \big (\widetilde{\varvec{\gamma }}^{(j-1)}\big )\right] \\&= \mathbb {E}\left[ \textbf{H}_n \big ({\varvec{\gamma }}_0\big ) + o_p(1)\right] \\&= \textbf{H}_n \big ({\varvec{\gamma }}_0\big ) + o(1), \end{aligned} \end{aligned}$$

where the last equality is implied by the standard regularity requirements (see, e.g., Guerrier et al. 2021) such as the compactness of $\varvec{\Gamma }$. Using the definition given in Eq. (6), we recall that $\textbf{V} = \lim _{n \rightarrow \infty } a_n \left\{ \textbf{A}^{\textrm{T}}\varvec{\Sigma }(\varvec{\gamma }_0)^{-1} \textbf{A} \right\} ^{-1}$ and thus, we have $\textbf{H}_n \big ({\varvec{\gamma }}_0\big ) = a_n \left\{ \textbf{A}^{\textrm{T}}\varvec{\Sigma }(\varvec{\gamma }_0)^{-1} \textbf{A} \right\} ^{-1} = \textbf{V} + o(1)$ and ${{\,\textrm{var}\,}}\left\{ \sqrt{a_n} \left( \widetilde{\textbf{x}}^{(j)} - \textbf{x}_0 \right) \right\} = \textbf{V} + o(1)$. From Eq. (6), we have that $\lim _{n \rightarrow \infty } \, {{\,\textrm{var}\,}}\left\{ \sqrt{a_n} \left( \widehat{\textbf{x}} - \textbf{x}_0 \right) \right\} = \textbf{V}$ and thus,

$$\begin{aligned}{} & {} \lim _{n \rightarrow \infty } \, {{\,\textrm{var}\,}}\left\{ \sqrt{a_n} \left( \widehat{\textbf{x}} - \textbf{x}_0 \right) \right\} - {{\,\textrm{var}\,}}\left\{ \sqrt{a_n} \left( \widetilde{\textbf{x}}^{(j)} - \textbf{x}_0 \right) \right\} = \textbf{0}, \end{aligned}$$

corresponding to Eq. (17).

## B. Further discussion on the Generalized Method of Wavelet Moments

As mentioned in Sect. 3.1, the GMWM estimator based on an estimator of $\textbf{x}_0$, say $\textbf{x}$, is defined as follows:

$$\begin{aligned} \widetilde{\varvec{\gamma }}\left( {\textbf {x}}\right) = \underset{\varvec{\gamma } \in \varvec{\Gamma }}{{{\,\text {argmin}\,}}}\; \left\{ \widehat{\varvec{\nu }}\left( {{\textbf {x}}}\right) - \varvec{\nu }(\varvec{\gamma })\right\} ^{\text {T}}\varvec{\Omega } \left\{ \widehat{\varvec{\nu }}\left( {{\textbf {x}}}\right) - \varvec{\nu }(\varvec{\gamma })\right\} , \end{aligned}$$

where $\varvec{\nu }(\varvec{\gamma })$ is the WV vector implied by the model and $\widehat{\varvec{\nu }}\left( {\textbf{x}}\right)$ is the estimated Haar WV computed on ${\varvec{\varepsilon }}\left( {\textbf{x}}\right)$. To define these quantities, we let

$$\begin{aligned}{} & {} W_{j,t}(\textbf{x}) = \sum _{l = 0}^{L_j - 1} h_{j,l} {\varvec{\varepsilon }}\left( {\textbf{x}}\right) _{t - l} \, , \end{aligned}$$

denote the wavelet coefficients associated with the (Haar) maximal overlap discrete wavelet transform (MODWT) wavelet decomposition of the time series (see, e.g., Percival and Walden 2000), where $(h_{j,t})$ is a known wavelet filter of length $L_j$ at scale $\tau _j = 2^j$, for $j = 1, \ldots , J$ and $J < \log _2(n)$. Based on this quantity, for $j = 1, \ldots , J$ the empirical WV at scale *j* is defined as

$$\begin{aligned} \nu _{j}^2(\textbf{x}) = {{\,\textrm{var}\,}}\{W_{j,t}(\textbf{x})\}, \end{aligned}$$

which corresponds to the variance of the wavelet coefficients. The vector $\widehat{\varvec{\nu }}\left( {\textbf{x}}\right)$ can then be expressed as $\varvec{\nu }(\textbf{x}) = [\nu _j^2(\textbf{x})]_{j = 1, \ldots , J}$. Several estimators have been proposed for the WV, we consider here the MODWT WV estimator proposed in Percival (1995), which enjoys desirable statistical properties. This estimator is simply defined as

B.2 $$\begin{aligned} \widehat{\nu }_{j}^2(\textbf{x}) = \frac{1}{M_{j}} \sum _{t=1}^{M_j} W_{j, t}^2(\textbf{x}) \,\,, \end{aligned}$$

where $M_j$ is the length of the wavelet coefficient process $(W_{j, t})$ at scale $\tau _j$. We define $\widehat{\varvec{\nu }}\left( {\textbf{x}}\right) = [\widehat{\nu }_{j}^2(\textbf{x})]_{j = 1, \ldots , J}$. A detailed introduction of the WV can be found in Percival and Walden (2000) and the references therein. Moreover, the theoretical properties of this estimator were further studied in for example Serroukh et al. (2000) and Guerrier et al. (2021) in which the conditions for its asymptotic properties are provided under different frameworks (such as those considered in this contribution).

In order to make the link between the WV and an assumed stationary (or intrinsically stationary) parametric model explicit, we have the following relation between the WV and the spectral density function (SDF):

B.3 $$\begin{aligned} \nu _{j}^2 ({\varvec{\gamma }}) = \int _{-1/2}^{1/2} |H_j(f)|^2 S(f) df \, , \end{aligned}$$

with *S*(*f*) denoting the theoretical SDF and $H_j(f)$ being the Fourier transform of the wavelet filters $(h_{j,t})$. In practice, computing  (B.3) is often difficult but the results of Zhang (2008) (see Eq. (11), which is here adapted to the WV instead of the Allan variance) provide the following result:

B.4 $$\begin{aligned}{} & {} \nu _{j}^2 ({\varvec{\gamma }}) = \frac{2}{\tau _j^2} \left[ \frac{\tau _j}{2} \left\{ 1 - \kappa \left( {\varvec{\gamma }}, \frac{\tau _j}{2}\right) \right\} \right. \nonumber \\{} & {} \quad \left. + \sum _{i = 1}^{\frac{\tau _j}{2} - 1} i \left\{ 2 \kappa \left( {\varvec{\gamma }}, \frac{\tau _j}{2} - i\right) - \kappa \left( {\varvec{\gamma }}, i\right) - \kappa \left( {\varvec{\gamma }}, \tau _j - i\right) \right\} \right] ,\nonumber \\ \end{aligned}$$

where $\tau _j = 2^j$ and $\kappa ({\varvec{\gamma }}, h)$ denotes the autocovariance function of $\textbf{Y}$, thus, for $h \in \mathbb {N}$ we have $\kappa ({\varvec{\gamma }}, h) = {{\,\textrm{cov}\,}}(\textbf{Y}_1, \textbf{Y}_h) = \varvec{\Sigma }({\varvec{\gamma }})_{1,h}$. Therefore, Eq. (B.4) provides a simple approach to compute the theoretical WV of nearly any (intrinsically) stationary parametric stochastic process. Indeed, the theoretical WV is a linear combination of the autocovariance function $\kappa ({\varvec{\gamma }}, h)$, which composes the elements of $\varvec{\Sigma }(\varvec{\gamma })$. In some sense, the vector $\varvec{\nu }(\varvec{\gamma })$ of dimension $J < \log _2(n)$ summarizes the main characteristics of the matrix $\varvec{\Sigma }(\varvec{\gamma })$ that can have up to *n* distinct elements (see, e.g., Percival and Walden 2000, Chapter 8, for more information)

Under suitable (mild) conditions (see Serroukh et al. 2000, see also Guerrier et al. 2021), we have

$$\begin{aligned}{} & {} \sqrt{M_j} \left( {\hat{\nu }}_j - \nu _j\right) {\overset{d}{\rightarrow }} \mathcal {N}(0, \sigma _j^2). \end{aligned}$$

Based on this result and using the asymptotic properties of the sum of squares of correlated random variables with zero mean and common variance, it can also be shown that

$$\begin{aligned} \frac{\eta {\hat{\nu }}_j}{\nu _j} {\overset{.}{\sim }}\; \chi ^2_{\eta }, \end{aligned}$$

where ${\overset{.}{\sim }}$ denotes “approximately follows” and $\eta$ is known as the “equivalent degrees of freedom” (see Percival and Walden 2000, Chapter 8). A possible and reasonable approximation of the latter is given by $\eta \approx {\hat{\eta }} = \max \left( 1, M_j/2^j \right)$. Thus, an approximate $100 \times (1-2\alpha )\%$ confidence interval for $\nu _j$ is given by

$$\begin{aligned}{} & {} \left[ \frac{{\hat{\eta }} {\hat{\nu }}_j}{Q_{{\hat{\eta }}}(1-\alpha )}, \frac{{\hat{\eta }} {\hat{\nu }}_j}{Q_{{\hat{\eta }}}(\alpha )}\right] , \end{aligned}$$

where $Q_{{\eta }}(\alpha )$ is the $100 \times \alpha \%$ percentage point of the $\chi ^2_{{\eta }}$ distribution. In this paper, the matrix $\widehat{\varvec{\Omega }}$ is computed using this approximation method and a reasonable choice for this quantity appears to be given by

B.5 $$\begin{aligned} \widehat{\varvec{\Omega }}_{i,j} = {\left\{ \begin{array}{ll} \left( \frac{{\hat{\eta }} {\hat{\nu }}_j}{Q_{{\hat{\eta }}}(1-\alpha )} - \frac{{\hat{\eta }} {\hat{\nu }}_j}{Q_{{\hat{\eta }}}(\alpha )}\right) ^{-2} &{} \text {if } i = j,\\ 0 &{} \text {if } i \ne j, \end{array}\right. } \end{aligned}$$

for $i,j = 1, \ldots , J$. This choice is motivated by the fact that $\widehat{\varvec{\Omega }}_{j,j}$ is readily available, completely nonparametric, and approximately inversely proportional to the variance of ${\hat{\nu }}_j$ (when the covariances among scales are ignored). This particular choice is also based on our empirical experience and desire for simplicity and is by no means optimal. Data-adaptive choices (e.g., based on resampling methods such as parametric bootstrap) have been considered in Guerrier et al. (2013, 2021), but we do not pursue this direction here.

## C. Additional simulation study: Setting A2

In this section, we present the results of an additional simulation study which considers Setting A2 (Figs. 9, 10).

## D. Additional simulation study: Setting B1

In this section, we present the results of an additional simulation study which considers Setting B1 (Figs. 11, 12).

## E. Additional simulation study: Setting B2

In this section, we present the results of an additional simulation study which considers Setting B2 (Figs. 13, 14).

## F. Confidence intervals with the GMWMX

We define the confidence interval used in Sect. 4.2. A $1-\alpha$ confidence interval for a parameter $\theta$ denoted $C_{n}=(f_{1}, f_{2})$ is the interval with random endpoints $f_{1}$ and $f_{2}$ where $f_{1}=f_{1}\left( X_{1}, \ldots , X_{n}\right)$ and $f_{2}=f_{2}\left( X_{1}, \ldots , X_{n}\right)$ are functions of the data such that

F.1 $$\begin{aligned} \Pr \left[ f_{1}(X_{1}, \ldots , X_{n}) \leqslant \theta \leqslant f_{2}(X_{1}, \ldots , X_{n}) \right] =1-\alpha .\nonumber \\ \end{aligned}$$

We call $1-\alpha$ the nominal coverage of the confidence interval.

An interesting property of the proposed estimators $\widetilde{{\varvec{\theta }}}_1$ and $\widetilde{{\varvec{\theta }}}_2$, which results from their consistency as well as the asymptotic distribution of $\widetilde{\textbf{x}}^{(1)}$ and $\widetilde{\textbf{x}}^{(2)}$, is that valid confidence intervals can be constructed for $\textbf{x}_0$. For $i = \{1, \ldots , p\}$, we let

F.2 $$\begin{aligned} \widetilde{\sigma }_{i,j}^2 = \left\{ \begin{array}{ll} \left[ \left( \textbf{A}^{\textrm{T}}\textbf{A} \right) ^{-1} \textbf{A}^{\textrm{T}}\varvec{\Sigma }\left( \widetilde{\varvec{\gamma }}^{(1)}\right) \textbf{A} \left( \textbf{A}^{\textrm{T}}\textbf{A} \right) ^{-1}\right] _{i,i} &{} \text{ if } j = 1, \\ \left[ \left\{ \textbf{A}^{\textrm{T}}\left[ \varvec{\Sigma }\left( \widetilde{\varvec{\gamma }}^{(2)}\right) \right] ^{-1} \textbf{A} \right\} ^{-1}\right] _{i,i} &{} \text{ if } j = 2. \end{array} \right. \nonumber \\ \end{aligned}$$

Using results of the asymptotic normality of $\widetilde{\textbf{x}}^{(1)}$ and $\widetilde{\textbf{x}}^{(2)}$ and Lemma 2.11 in Van der Vaart (2000), it can be shown that for $j \in \{1,2\}$ as well as for all $\alpha \in (0, 0.5)$ and for all $i \in \{1, \ldots , p\}$ we have

$$\begin{aligned} \lim _{n \rightarrow \infty } \; \Pr \left[ \left( \textbf{x}_0\right) _i \in \left\{ \big (\widetilde{\textbf{x}}^{(j)} \big )_i \pm z_{1-\alpha /2} \widetilde{\sigma }_{i,j} \right\} \right] = 1-\alpha , \end{aligned}$$

where $z_{1-\alpha /2}$ denotes the $1-\alpha /2$ quantile of the standard normal distribution. This standard result demonstrates the asymptotic validity of confidence intervals constructed with the proposed approach.

## G. Additional information

See Fig. 15 and Tables 1, 2, 3.

**Table 1:** Estimated North velocities for the 33 stations considered in the case study using the GMWMX-1, GMWMX-2, the MLE as implemented in Hector and PBO velocity estimates

	GMWMX-1		GMWMX-2		MLE (Hector)		PBO	
	${\hat{b}}$	${\hat{\sigma }}_{b}$	${\hat{b}}$	${\hat{\sigma }}_{b}$	${\hat{b}}$	${\hat{\sigma }}_{b}$	${\hat{b}}$	${\hat{\sigma }}_{b}$
*North* (mm/y)								
ANP5	4.12	0.08	4.12	0.09	4.06	0.10	4.11	0.10
ANP6	4.17	0.06	4.16	0.06	4.14	0.05	4.13	0.10
ASUB	2.91	0.04	2.95	0.04	2.88	0.12	2.43	0.20
BACO	4.01	0.06	4.04	0.06	4.00	0.05	4.09	0.10
BLA1	3.07	0.04	3.07	0.04	3.09	0.06	3.12	0.11
COLA	4.38	0.11	4.57	0.11	4.35	0.12	4.14	0.22
DNRC	4.51	0.03	4.51	0.03	4.50	0.04	3.87	0.23
DOBS	3.20	0.09	3.23	0.08	3.27	0.08	3.32	0.14
DRV5	5.32	0.07	5.38	0.07	5.41	0.08	5.42	0.12
DRV6	4.20	0.02	4.21	0.02	4.22	0.04	4.20	0.09
GAST	2.86	0.07	2.87	0.07	2.89	0.08	2.85	0.10
HIPT	3.23	0.07	3.24	0.07	3.22	0.07	3.29	0.10
HNPT	4.35	0.08	4.36	0.09	4.36	0.09	4.12	0.14
KNS5	3.11	0.14	3.14	0.14	3.05	0.13	3.29	0.13
KNS6	3.23	0.16	3.37	0.16	3.30	0.15	3.42	0.19
KYTL	2.33	0.06	2.32	0.06	2.33	0.05	2.12	0.16
NBR5	3.59	0.05	3.62	0.05	3.64	0.07	3.69	0.13
NBR6	3.90	0.03	3.90	0.03	3.90	0.05	3.95	0.08
NCCH	3.71	0.07	3.77	0.07	3.73	0.06	3.71	0.11
NCJA	3.60	0.05	3.57	0.05	3.60	0.04	3.55	0.09
NCSW	2.41	0.04	2.40	0.04	2.42	0.07	2.19	0.12
NCWH	3.50	0.07	3.48	0.07	3.48	0.06	3.50	0.11
NJTW	4.45	0.04	4.39	0.04	4.41	0.04	4.42	0.08
OHLI	2.30	0.04	2.30	0.04	2.27	0.05	2.16	0.12
PAFU	2.88	0.06	2.90	0.06	2.88	0.05	2.84	0.10
SCGP	2.74	0.06	2.75	0.06	2.76	0.07	2.79	0.11
SCWT	2.26	0.02	2.29	0.02	2.29	0.07	2.56	0.13
UVFM	3.47	0.05	3.57	0.05	3.52	0.05	3.47	0.09
WVBU	3.38	0.07	3.48	0.07	3.45	0.08	3.50	0.11
WVHU	2.25	0.03	2.24	0.03	2.23	0.05	2.34	0.15
WVRA	1.81	0.06	1.79	0.06	1.82	0.07	1.83	0.07
YORK	4.24	0.04	4.22	0.04	4.22	0.03	4.20	0.08
ZDC1	3.68	0.06	3.65	0.06	3.62	0.06	3.34	0.15

The estimated velocity for each method is denoted by ${\hat{\mu }}$ and ${\hat{\sigma }}_b$ denotes its estimated uncertainty (standard error)

**Table 2:** Estimated East velocities for the 33 stations considered in the case study using the GMWMX-1, GMWMX-2, the MLE implemented in Hector and PBO velocity estimates

	GMWMX-1		GMWMX-2		MLE (Hector)		PBO	
	${\hat{b}}$	${\hat{\sigma }}_{b}$	${\hat{b}}$	${\hat{\sigma }}_{b}$	${\hat{b}}$	${\hat{\sigma }}_{b}$	${\hat{b}}$	${\hat{\sigma }}_{b}$
*East* (mm/y)								
ANP5	$-$ 14.50	0.06	$-$ 14.53	0.07	$-$ 14.50	0.10	$-$ 14.50	0.10
ANP6	$-$ 14.83	0.03	$-$ 14.83	0.03	$-$ 14.84	0.09	$-$ 14.84	0.09
ASUB	$-$ 14.14	0.04	$-$ 14.12	0.04	$-$ 13.84	0.16	$-$ 13.84	0.16
BACO	$-$ 14.70	0.03	$-$ 14.70	0.03	$-$ 14.43	0.12	$-$ 14.43	0.12
BLA1	$-$ 14.33	0.05	$-$ 14.33	0.05	$-$ 14.34	0.10	$-$ 14.34	0.10
COLA	$-$ 13.48	0.11	$-$ 13.61	0.11	$-$ 13.16	0.12	$-$ 13.16	0.12
DNRC	$-$ 14.56	0.21	$-$ 14.57	0.21	$-$ 14.87	0.17	$-$ 14.87	0.17
DOBS	$-$ 13.92	0.09	$-$ 13.93	0.09	$-$ 13.91	0.10	$-$ 13.91	0.10
DRV5	$-$ 13.58	0.06	$-$ 13.54	0.06	$-$ 13.47	0.13	$-$ 13.47	0.13
DRV6	$-$ 14.27	0.06	$-$ 14.29	0.06	$-$ 14.23	0.09	$-$ 14.23	0.09
GAST	$-$ 13.56	0.10	$-$ 13.54	0.10	$-$ 14.11	0.11	$-$ 14.11	0.11
HIPT	$-$ 13.83	0.07	$-$ 13.84	0.07	$-$ 13.90	0.10	$-$ 13.90	0.10
HNPT	$-$ 14.52	0.12	$-$ 14.73	0.12	$-$ 14.48	0.11	$-$ 14.48	0.11
KNS5	$-$ 12.84	0.14	$-$ 12.76	0.15	$-$ 12.65	0.19	$-$ 12.65	0.19
KNS6	$-$ 13.44	0.23	$-$ 13.33	0.23	$-$ 13.04	0.27	$-$ 13.04	0.27
KYTL	$-$ 14.32	0.07	$-$ 14.34	0.07	$-$ 14.21	0.11	$-$ 14.21	0.11
NBR5	$-$ 13.07	0.03	$-$ 13.10	0.03	$-$ 13.11	0.13	$-$ 13.11	0.13
NBR6	$-$ 13.53	0.06	$-$ 13.53	0.06	$-$ 13.49	0.07	$-$ 13.49	0.07
NCCH	$-$ 13.44	0.05	$-$ 13.46	0.05	$-$ 13.34	0.10	$-$ 13.34	0.10
NCJA	$-$ 12.87	0.04	$-$ 12.85	0.04	$-$ 12.80	0.10	$-$ 12.80	0.10
NCSW	$-$ 13.75	0.04	$-$ 13.77	0.04	$-$ 13.66	0.11	$-$ 13.66	0.11
NCWH	$-$ 13.03	0.07	$-$ 13.02	0.07	$-$ 12.97	0.10	$-$ 12.97	0.10
NJTW	$-$ 14.93	0.04	$-$ 14.93	0.04	$-$ 14.80	0.08	$-$ 14.80	0.08
OHLI	$-$ 15.00	0.02	$-$ 15.00	0.02	$-$ 14.95	0.10	$-$ 14.95	0.10
PAFU	$-$ 14.90	0.04	$-$ 14.87	0.04	$-$ 14.90	0.09	$-$ 14.90	0.09
SCGP	$-$ 13.76	0.06	$-$ 13.77	0.06	$-$ 13.85	0.12	$-$ 13.85	0.12
SCWT	$-$ 13.26	0.08	$-$ 13.26	0.08	$-$ 13.48	0.17	$-$ 13.48	0.17
UVFM	$-$ 14.36	0.01	$-$ 14.38	0.01	$-$ 14.35	0.09	$-$ 14.35	0.09
WVBU	$-$ 14.75	0.02	$-$ 14.74	0.02	$-$ 14.23	0.11	$-$ 14.23	0.11
WVHU	$-$ 14.67	0.09	$-$ 14.71	0.09	$-$ 14.40	0.14	$-$ 14.40	0.14
WVRA	$-$ 14.54	0.06	$-$ 14.58	0.06	$-$ 14.33	0.06	$-$ 14.33	0.06
YORK	$-$ 14.52	0.05	$-$ 14.51	0.05	$-$ 14.51	0.07	$-$ 14.51	0.07
ZDC1	$-$ 15.11	0.01	$-$ 15.11	0.01	$-$ 15.12	0.08	$-$ 15.12	0.08

The estimated velocity for each method is denoted by ${\hat{\mu }}$ and ${\hat{\sigma }}_b$ denotes its estimated uncertainty (standard error)

**Table 3:** Estimated crustal uplift for the 33 stations considered in the case study using the GMWMX-1, GMWMX-2, the MLE implemented in Hector and PBO velocity estimates

	GMWMX-1		GMWMX-2		MLE (Hector)		PBO	
	${\hat{b}}$	${\hat{\sigma }}_{b}$	${\hat{b}}$	${\hat{\sigma }}_{b}$	${\hat{b}}$	${\hat{\sigma }}_{b}$	${\hat{b}}$	${\hat{\sigma }}_{b}$
*Up* (mm/y)								
ANP5	$-$ 3.24	0.22	$-$ 3.20	0.22	$-$ 3.25	0.24	$-$ 3.22	0.32
ANP6	$-$ 2.67	0.23	$-$ 2.70	0.23	$-$ 2.60	0.26	$-$ 2.75	0.25
ASUB	$-$ 0.78	0.20	$-$ 0.72	0.20	$-$ 0.68	0.28	$-$ 2.42	1.58
BACO	$-$ 1.44	0.32	$-$ 1.47	0.32	$-$ 1.47	0.33	$-$ 1.66	0.24
BLA1	$-$ 1.56	0.34	$-$ 1.54	0.34	$-$ 1.39	0.35	$-$ 1.54	0.30
COLA	$-$ 2.05	0.15	$-$ 2.00	0.15	$-$ 2.07	0.24	$-$ 3.99	0.66
DNRC	$-$ 2.85	0.19	$-$ 2.82	0.19	$-$ 2.89	0.22	$-$ 2.55	0.49
DOBS	$-$ 1.06	0.28	$-$ 0.88	0.28	$-$ 0.93	0.30	$-$ 0.99	0.46
DRV5	$-$ 2.69	0.18	$-$ 2.84	0.18	$-$ 2.80	0.20	$-$ 2.61	0.26
DRV6	$-$ 3.25	0.16	$-$ 3.32	0.16	$-$ 3.32	0.17	$-$ 3.36	0.26
GAST	$-$ 0.88	0.22	$-$ 0.62	0.22	$-$ 0.73	0.25	$-$ 0.77	0.41
HIPT	$-$ 0.99	0.27	$-$ 0.96	0.27	$-$ 0.87	0.30	$-$ 1.87	0.31
HNPT	$-$ 2.65	0.23	$-$ 2.75	0.23	$-$ 2.61	0.25	$-$ 2.64	0.19
KNS5	$-$ 3.54	0.18	$-$ 3.57	0.18	$-$ 3.57	0.20	$-$ 3.72	0.40
KNS6	$-$ 2.59	0.16	$-$ 2.80	0.16	$-$ 2.70	0.20	$-$ 2.61	0.41
KYTL	$-$ 1.55	0.25	$-$ 1.49	0.25	$-$ 1.50	0.28	$-$ 3.12	1.35
NBR5	$-$ 1.81	0.13	$-$ 1.84	0.13	$-$ 1.84	0.15	$-$ 2.02	0.31
NBR6	$-$ 1.80	0.13	$-$ 1.77	0.13	$-$ 1.79	0.16	$-$ 1.93	0.18
NCCH	$-$ 1.80	0.17	$-$ 1.72	0.17	$-$ 1.72	0.18	$-$ 2.08	0.37
NCJA	$-$ 1.02	0.24	$-$ 0.84	0.24	$-$ 0.96	0.25	$-$ 1.16	0.29
NCSW	0.02	0.21	$-$ 0.01	0.21	0.05	0.26	$-$ 0.13	0.40
NCWH	$-$ 2.08	0.20	$-$ 1.81	0.20	$-$ 1.97	0.26	$-$ 2.47	0.39
NJTW	$-$ 2.50	0.18	$-$ 2.44	0.18	$-$ 2.46	0.19	$-$ 2.35	0.25
OHLI	$-$ 2.46	0.26	$-$ 2.35	0.26	$-$ 2.51	0.27	$-$ 3.33	0.96
PAFU	$-$ 1.78	0.27	$-$ 1.88	0.27	$-$ 1.79	0.32	$-$ 2.99	0.46
SCGP	$-$ 2.57	0.19	$-$ 2.66	0.19	$-$ 2.51	0.26	$-$ 2.95	0.44
SCWT	$-$ 1.12	0.16	$-$ 1.17	0.16	$-$ 1.21	0.22	$-$ 2.20	0.51
UVFM	$-$ 1.29	0.26	$-$ 1.10	0.26	$-$ 1.31	0.27	$-$ 1.54	0.24
WVBU	$-$ 1.92	0.23	$-$ 1.72	0.23	$-$ 1.86	0.27	$-$ 1.59	0.29
WVHU	$-$ 1.71	0.34	$-$ 1.35	0.34	$-$ 1.57	0.34	$-$ 3.04	1.20
WVRA	$-$ 1.66	0.29	$-$ 1.40	0.29	$-$ 1.55	0.28	$-$ 1.99	0.19
YORK	$-$ 1.61	0.22	$-$ 1.59	0.22	$-$ 1.57	0.21	$-$ 1.53	0.19
ZDC1	$-$ 1.55	0.25	$-$ 1.67	0.25	$-$ 1.64	0.23	$-$ 1.70	0.21

The estimated velocity for each method is denoted by ${\hat{\mu }}$ and ${\hat{\sigma }}_b$ denotes its estimated uncertainty (standard error)
